# Semi-supervised learning for automated perineural invasion detection in multi-organ H&E whole slide images

**DOI:** 10.1093/bioinformatics/btag461

**Published:** 2026-08-21

**Authors:** A Alkhan, M Lynch, E J Ryan, M Lavelle, A C Culhane

**Affiliations:** School of Medicine, Limerick Digital Cancer Research, and Health Research Institute, University of Limerick, Co Limerick, V94 T9PX, Ireland; School of Medicine, Limerick Digital Cancer Research, and Health Research Institute, University of Limerick, Co Limerick, V94 T9PX, Ireland; Department of Biological Science, Limerick Digital Cancer Research Centre, Health Research Institute, University of Limerick, Co Limerick, V94 T9PX, Ireland; Pathology Department, University Hospital Limerick, Co Limerick, V94 F858, Ireland; School of Medicine, Limerick Digital Cancer Research, and Health Research Institute, University of Limerick, Co Limerick, V94 T9PX, Ireland

## Abstract

**Motivation:**

Perineural invasion (PNI) is an important pathological phenotype associated with poor prognosis in multiple malignancies. The primary detection method is visual inspection of whole slide images (WSIs), which is labor-intensive, time-consuming, subjective, and prone to high inter-observer variability. Developing reliable, accurate deep learning models for PNI detection is constrained by the lack of pixel-level annotated WSIs.

**Results:**

We evaluated four backbone architectures and two different approaches to improve PNI detection in a multi-organ dataset of colon, prostate, and pancreatic adenocarcinomas. We report three key findings. First, two pathology-pretrained foundation models, Virchow-2 and UNI, substantially outperformed ImageNet-pretrained CNNs (EfficientNet-B3, ConvNeXt-2), with distinct baseline error profiles reflecting differences in pretraining-data composition. Second, a data curation strategy driven by confidence-based pseudo-labelling (threshold *P* > .9) with human-in-the-loop review expanded the dataset from 262 to 352 WSIs, yielding a 12.4% relative F1 improvement (0.740 to 0.832) and a 55.5% reduction in false positives per slide; an ablation attributed 70% of the F1 gain to data volume and 41% of the FP reduction to benign-class curation. Third, per-organ analysis revealed that the primary driver of this improvement was not data volume alone but the targeted annotation enrichment of underrepresented morphologies in adjacent-normal and benign tissue, including desmoplastic stroma, crypts, and small blood vessels, that had been a systematic source of false positive predictions across tissue types.

**Availability:**

Our implementation is available at https://github.com/AhmadAlkhan/PNI_SSL.

## 1 Introduction

**Figure 1:**
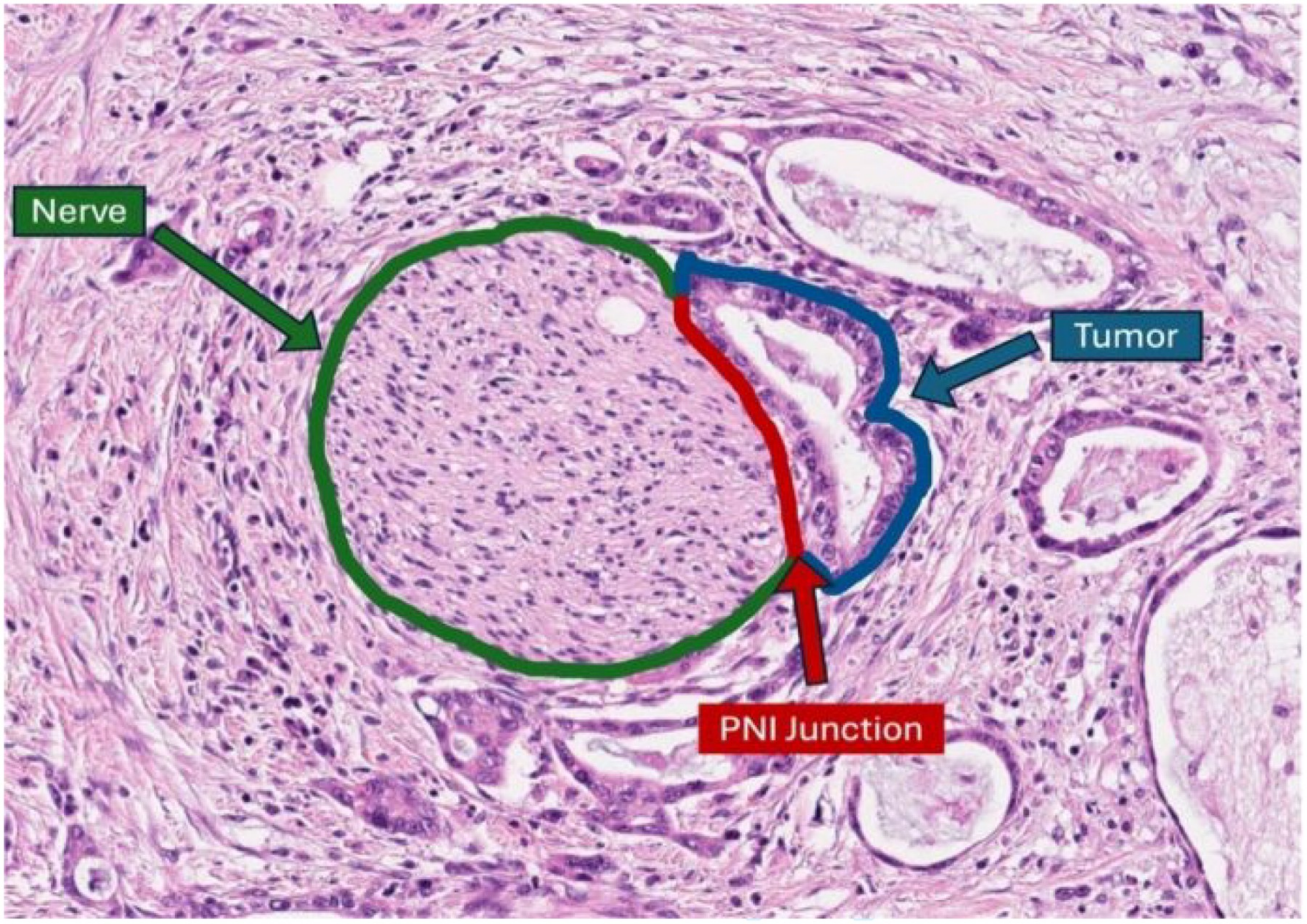
Image of a haematoxylin and eosin-stained tissue section of pancreatic cancer in which tumour (blue), peripheral nerve (green) and PNI junction (red) are highlighted.

Perineural invasion (PNI) is defined as the infiltration of cancer cells into the perineurial space or the direct juxtaposition of tumour cells against a nerve involving at least 33% of its circumference (Fig. 1) (Liebig *et al*. 2009a). This pathological feature is an important prognostic indicator across several malignancies including colon, prostate, pancreatic, head and neck cancers. In pancreatic ductal adenocarcinoma, PNI is pervasive, occurring in 70–100% of cases and correlating strongly with early tumour recurrence, neuropathic pain, and diminished survival rates (Schorn *et al.* 2017). Similarly, in prostate cancer, PNI detected in needle biopsies serves as an independent predictor of extraprostatic extension and bone metastasis (Delahunt *et al.* 2020). In colorectal cancer, PNI incidence ranges from 9 to 30% and significantly impacts treatment decisions and adjuvant therapy recommendations (Wang *et al.* 2024). Despite its clinical weight, PNI is routinely collected in only a subset of cancer types (Chen *et al.* 2019). Detection is carried by microscopic inspection that is labour intensive, and prone to inter-observer variability, with inter-observer discordance rates reported as high as 26% in difficult cases (Van Wyk *et al.* 2017).

Deep learning (DL) has emerged as a powerful tool in digital pathology enabling automated analysis of complex histopathological images. Combination of digital pathology DL with spatial “omics” is an emerging area in bioinformatics. High quality pathology annotations are critical for spatial biology and interpretation of single cell spatial “omics” because these provide the morphological context, alignment framework, and computational infrastructure that make spatial-omics interpretable and scalable (Li *et al.* 2025). However, the availability and success of supervised DL digital pathology models rely on large-scale, expert-curated, pixel-level annotations. In the context of PNI, acquiring such datasets is challenging. PNI is not routinely annotated as part of care, and pathologist manual curation is required (Liebig *et al*. 2009a). As a result, there is a scarcity of expert-annotated datasets, and this remains the primary bottleneck preventing the clinical deployment of PNI detection systems. PNI regions are often minute relative to the gigapixel resolution of whole slide images (WSIs), creating a “needle in a haystack” problem. Furthermore, there is morphological variability of both tumour cells and nerve fibres across different organs (e.g., colon vs. pancreas) complicating the training of generalizable models. To overcome the data scarcity bottleneck, we propose a semi-supervised learning (SSL) framework that synergises the representational power of pathology-specific foundation models with a human-in-the-loop pseudo-labelling strategy. Our main contributions are threefold. First, we benchmark two pathology-pretrained foundation models (Virchow-2 and UNI) (Chen *et al.* 2024, Zimmermann *et al.* 2024) against ImageNet-pretrained CNN architectures (EfficientNet-B3, ConvNeXt-2) for PNI detection. Second, we apply confidence-thresholded pseudo-labelling combined with targeted human-in-the-loop curation to expand PNI training data from 262 to 352 WSIs across multi-organ cancers. Third, we demonstrate that the SSL framework handles domain shifts across colon, prostate, and pancreatic tissues.

### 1.1 PNI detection methods

Recent attempts to develop automated PNI detection relied on engineered features and standard Convolutional Neural Networks (CNNs). Kartasalo *et al.* demonstrated a deep neural network capable of detecting PNI in prostate biopsies, trained on 80 000 biopsy cores from STHLM3 trial, with an AUC of 0.98, though their method was restricted to a single organ (Kartasalo *et al.* 2022). The Pathology AI Platform Challenge (PAIP 2021) expanded this scope, calling for multi-organ solutions across colon, prostate, and pancreas Top-performing entry achieved F1-score of 0.597 (PAIP 2021), however, they have utilised heavy data augmentation and ensemble methods but highlighted the fragility of models trained on limited annotated cohorts. These approaches typically required extensive pixel-level annotations by expert pathologists, limiting scalability. to new datasets and organs.

### 1.2 Foundation models in pathology

The computational pathology field is currently undergoing a paradigm shift from ImageNet-pretrained CNNs to domain-specific foundation models trained on vast volumes of data. Virchow (Vorontsov *et al.* 2024) and its successor Virchow-2 (Zimmermann *et al.* 2024) are leading this frontier. Virchow-2 is a Vision Transformer (ViT-H/14) with 632 million parameters, pretrained on 3.1 million WSIs using a modified DINOv2 algorithm with pathology-specific adaptations. Unlike general vision models, Virchow-2 was exposed to diverse staining protocols (H&E and immunohistochemistry) and mixed magnifications (5×, 10×, 20×, 40×) during pretraining, allowing it to generate robust patch embeddings that generalise well to downstream tasks with minimal fine-tuning. The training data included mostly cancer pathology tissue from several institutions from different continents. Breast was the most represented organ. About 7% of the training data was colon and achieving a Pearson correlation in molecular subtyping of 0.127, prostate made up 6.5% of the data, and achieved 0.383, and pancreas made up less than 1% and achieved 0.425. UNI (Chen *et al.* 2024) is a ViT-L/16 (307M parameters) general purpose pretrained on 100 426 diagnostic H&E WSIs spanning 20 tissue types, including normal tissue, cancerous tissue and other pathologies. Heart was the most represented organ.

### 1.3 Semi-supervised learning in medical imaging

Semi-supervised learning (SSL) mitigates the need for massive, labelled datasets by leveraging unlabelled data during learning. The two dominant SSL paradigms are consistency regularisation and pseudo-labelling (van Engelen and Hoos 2020). FixMatch (Sohn *et al.* 2020) combine these approaches, enforcing prediction consistency across augmentations above a high confidence threshold (typically 0.95). Histopathology frameworks including CLAM (Lu *et al.* 2021) and Semi-His-Net (Jiang *et al.* 2022) have applied weak supervision for tumour classification, reporting that pseudo-label quality matters more than quantity. Applying threshold-based SSL to PNI detection remains unexplored, particularly regarding the trade-off between pseudo-label quantity and the risk of not capturing benign tissue mimicking PNI, such as desmoplastic stroma in pancreatic tissue, crypts in colorectal tissue and small blood vessels. From SSL paradigms, we adopted pseudo-labelling (Lee 2013) rather than consistency regularisation (Tarvainen and Valpola 2017) or hybrid approaches, FixMatch (Sohn *et al.* 2020) because it produces explicit categorical predictions that can be directly reviewed and corrected by a pathologist, enabling targeted curation of the class.

## 2 Methods

### 2.1 Dataset and preprocessing

We utilised the dataset from the PAIP (2021) Challenge and the WISEPAIP repository (SNUH 2021) as the teacher part of training, as it has trusted high quality labels. Annotations included PNI, tumour, nerve, and benign.

- Labelled Data (LD): 150 WSIs comprising 50 colon, 50 prostate, and 50 pancreatic adenocarcinomas with pixel-level annotations for PNI,
- Bounding box-level labelled Data (BLD): 112 additional PDAC WSIs were obtained from WISEPAIP repository. The BLD data were left out from the SSL approach but were used to train both models.
- Unlabelled Data (UD): 90 unannotated WSIs utilised for pseudo-label generation. These data were used for the SSL approach to obtain predicted pseudo-labels.

All WSIs were tiled into 224 × 224-pixel patches at 20× and 5× magnification. Background patches were discarded using Otsu’s thresholding (Otsu 1979) applied to grayscale images with binary inversion to separate white background from tissue. The 352 WSIs were divided into 65% training, 23% validation, and 12% testing splits. Crucially, the holdout test set comprised 45 WSIs drawn exclusively from the expert-annotated labelled data (LD) and selected randomly, with 15 WSIs per organ (colon, prostate, and pancreas), ensuring that evaluation was performed only against high-quality ground truth annotations. Pseudo-labelled WSIs and bounding box-labelled WSIs (BLD) were used only for training and validation, never for testing. This design prevents any inflation of test performance due to evaluation on pseudo-labelled data.

### 2.2 Model architectures

**Figure 2:**
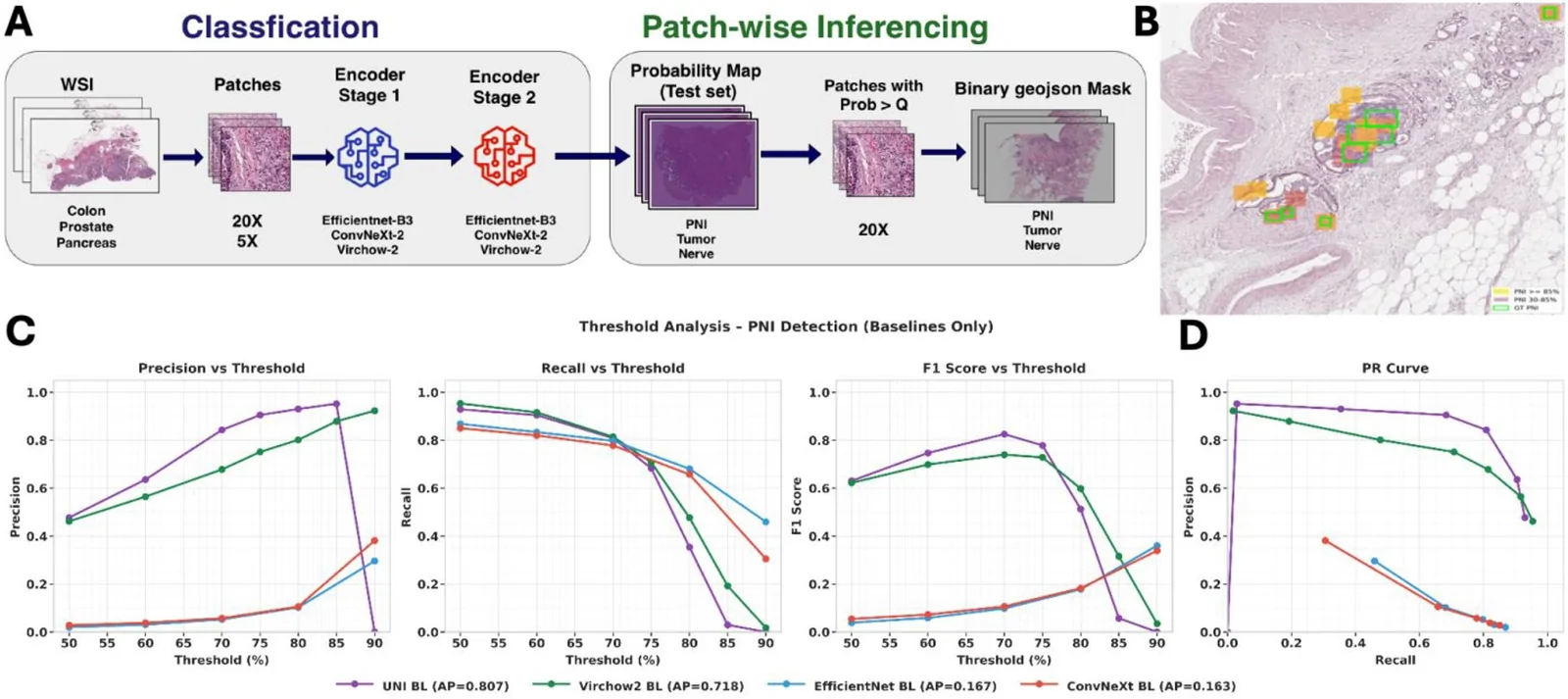
Model architecture and classification performance. (A) Two-stage pipeline: patches extracted at 20× and 5× magnification are classified into PNI, Tumour, and Nerve; patches exceeding threshold Q (0.5–0.9) at 20× are retained to produce binary GeoJSON masks. (B) Activation map from a colon case (slide 7). Green box: ground truth; yellow: PNI probability ≥85%; violet: 30–85%. (C) PNI detection performance of the baseline approach across probability thresholds for Virchow-2 (green), UNI (purple), EfficientNet-B3 (blue), and ConvNeXt-2 (red), showing precision, recall, and F1-score. (D) precision-recall curves for PNI detection under the baseline approach for all four architectures.

We benchmarked four distinct architectures (Fig. 2A):

- EfficientNet-B3: A compound-scaled CNN baseline (∼12M parameters) pretrained on ImageNet (Tan and Le 2019).
- ConvNeXt-2: A transformer-style macro designs and masked autoencoder pretrained on ImageNet (Woo *et al.* 2023).
- Virchow-2: A large-scale Vision Transformer (ViT-H) with 632M parameters, pretrained on 3.1M pathology WSIs via DINOv2 (Zimmermann *et al.* 2024).
- UNI: A Vision Transformer (ViT-L/16) with 307M parameters, pretrained via DINOv2 on 100K H&E WSIs (Chen *et al.* 2024).

All architectures followed a two-stage training protocol. Stage 1 trained on all classes. Stage 2 fine-tuned binary refinement classifiers per class group to filter stage 1 false positives, using layer-wise learning rate decay (factor 0.75) to preserve pretrained representations in earlier layers. Model hyperparameters, training strategy, evaluation metric definitions, computational cost analysis (Tables S1–S3), and summary per-class detection results for all architectures under all conditions (Tables S5 and S6a and b) are provided in Supplementary Materials.

### 2.3 Training conditions

To disentangle pseudo-label volume from benign-class curation, we trained each backbone under three conditions: Baseline (262 WSIs, no SSL); Ablation (352 WSIs, no curation); and SSL (352 WSIs, pseudo-labels with manual benign curation, expanding annotations from 2390 to 2680 bounding boxes and from 24 × 10^9^ to 56 × 10^9^ pixels).

### 2.4 Semi-supervised learning pipeline and benign class curation

**Figure 3:**
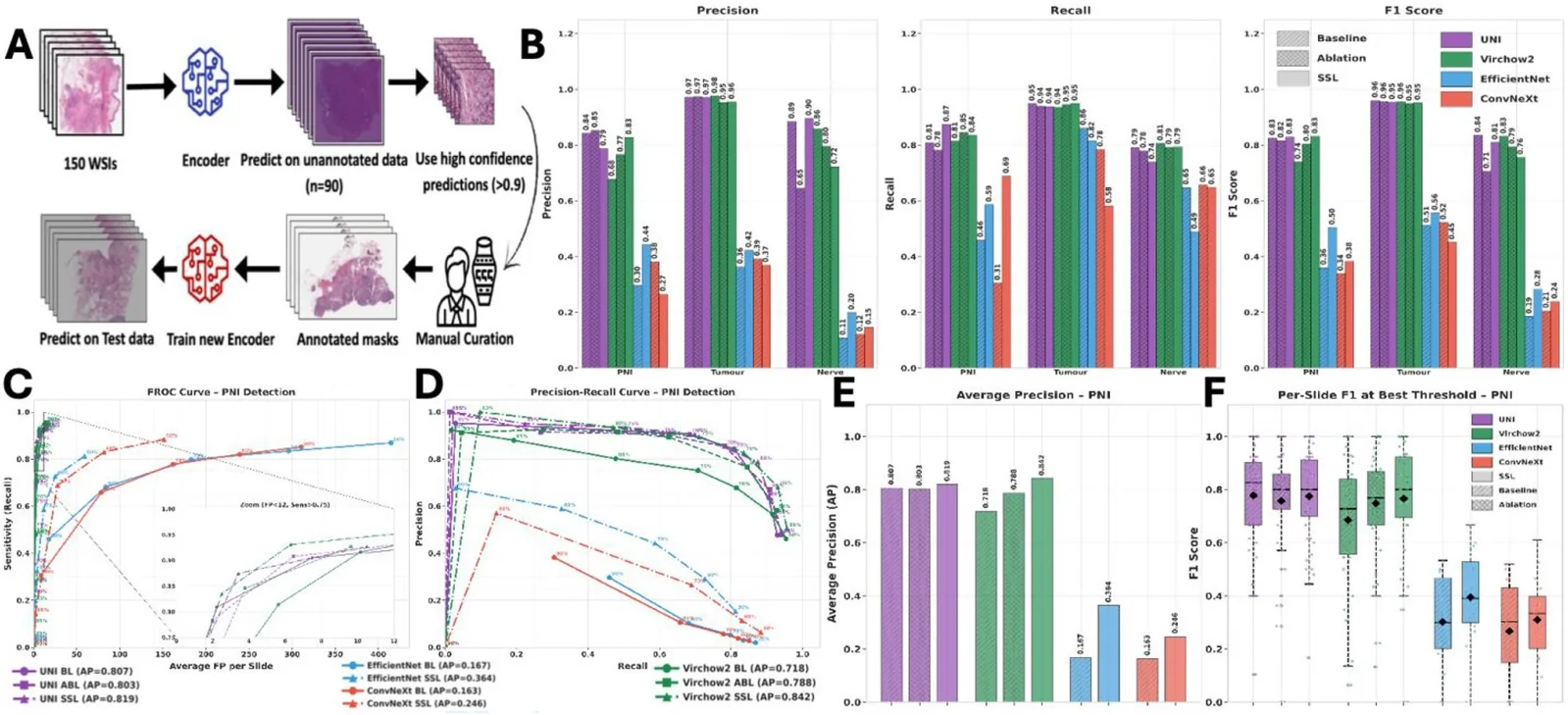
SSL pipeline and model performance. (A) SSL pipeline: teacher model trained on 150 annotated WSIs generates pseudo-labels on 90 unannotated slides (threshold >0.9), followed by manual curation and student retraining on 352 WSIs. (B) Per-class (PNI, Tumour, Nerve) precision, recall, and F1-score at best threshold for all 10 model configurations UNI (purple), Virchow-2 (green), EfficientNet-B3 (blue), ConvNeXt-2 (red) trained with Baseline (hatched), Ablation (cross-hatched) or SSL (solid) conditions (C) Free-response receiver operating characteristic (FROC) curves showing sensitivity against average FP per slide for PNI detection across all 10 configurations where training condition is denoted by marker shape (circle = Baseline, square = Ablation, triangle = SSL); inset is zoomed view at FP < 18. (D) Precision-recall curves with confidence thresholds annotated (50%–90%) for all 10 configurations. (E) Average precision and (F) Per-slide F1 boxplots at each model’s best threshold on holdout test set (n = 45) for all 10 configurations. The mean (diamond) per slide F1 at best threshold was lower than median (line) and is indicated on the boxplot.

Our proposed framework operates in three stages (Fig. 3A). In the first stage, a “Teacher” model was trained on the 150 expert-annotated WSIs. In the second stage, the Teacher generated predictions on the 90 unlabelled WSIs, and only patches where the maximum class probability exceeded 0.9 were retained as pseudo-labels. This threshold was selected based on findings from FixMatch (Sohn *et al.* 2020) that pseudo-label quality is more critical than quantity. In the third stage, the curated pseudo-labels were combined with the original annotated masks to create an expanded training set, increasing the dataset from 150 to 240 WSIs. An additional 112 PDAC WSIs (BLD) from the WISEPAIP repository were added, bringing the total to 352 WSIs. A new encoder was then trained on this expanded dataset. Patches with high predicted PNI probability were manually reviewed as part of this stage, serving three functions: (i) removing false positive predictions arising from non-neoplastic structures that mimic nerve morphology, (ii) reannotating the benign class to include hard-to-classify morphologies that were underrepresented in the initial training set, and (iii) validating high-confidence PNI predictions to ensure label fidelity in the expanded dataset.

## 3 Results

We first compare the four architectures and show that both pathology-pretrained models (UNI and Virchow-2) outperform ImageNet CNNs, with distinct error profiles (Section 3.1). We decompose the SSL gain into a data-volume component and a curation component (Section 3.2). Finally, we show that curation benefit is driven by correcting organ-specific FP patterns in the benign class (Section 3.3).

### 3.1 Pathology foundation models outperform ImageNet CNNs

To isolate the effect of model architecture from the semi-supervised expansion, we first evaluated all four backbone architectures trained on the baseline dataset (262 WSIs: 150 PAIP + 112 WISEPAIP) and show domain-specific foundation models have clear performance advantage over ImageNet-pretrained CNN architectures (Table 1), confirming that foundation models pretrained on massive histopathology datasets learn feature representations that are significantly more discriminative for subtle features like PNI than ImageNet-pretrained networks. UNI achieved the strongest baseline (F1 = 0.826, 2.22 FP/slide), exceeding Virchow-2 (F1 = 0.740, 5.64 FP/slide). Virchow-2’s residual baseline errors were concentrated in pancreatic tissue (10.4 FP/slide), where benign structures, particularly desmoplastic stroma, were misclassified, which motivated the curation strategy in Section 3.3.

**Table 1:** Performance comparison of models on PNI detection (holdout test set, n = 45) trained on 262 WSIs without SSL expansion (Baseline).

Model	Precision	Recall	F1	AP*	FP*/Slide
UNI	**0.843**	0.809	**0.826**	**0.807**	**2.22**
Virchow-2	0.678	**0.814**	0.740	0.718	5.64
EfficientNet-B3	0.296	0.460	0.360	0.167	18
ConvNeXt-2	0.381	0.305	0.339	0.163	8

* FP = false positive, AP = average precision. Bold text = Highest value within the column.

### 3.2 Semi-supervised learning (SSL) improves detection

We evaluated expanding the training data from 262 to 352 WSIs through confidence-based pseudo-labelling (*P* > .9) and human-in-the-loop curation. Table 2 summarises the performance of the four backbone architectures on the holdout test set (n = 45) after training with the SSL-expanded dataset. All metrics are reported at each model’s optimal operating threshold, determined by maximising F1-score on the validation set. Virchow-2 SSL achieved the highest F1 (0.832), narrowly above UNI SSL (0.829). Notably, ConvNeXt-2 exhibited the lowest precision (0.265) despite achieving a high recall (0.689), suggesting that its masked autoencoder pretraining strategy, while effective at capturing PNI-relevant features, produces broadly sensitive predictions that generate many false alarms.

**Table 2:** Performance comparison of models on PNI detection (holdout test set, n = 45) trained on 352 WSIs with SSL expansion.

Model	Precision	Recall	F1	AP	FP/Slide
Virchow-2	**0.828**	0.835	**0.832**	**0.872**	**2.51**
UNI	0.788	**0.874**	0.829	0.846	3.42
EfficientNet-B3	0.442	0.586	0.504	0.364	12
ConvNeXt-2	0.265	0.689	0.383	0.246	27

#### 3.2.1 Quantifying the SSL benefit: ablation

To isolate the contribution of pseudo-label volume from benign-class curation, we evaluated an Ablation condition (352 WSIs with pseudo-labels but no manual curation). The Ablation condition decomposes the SSL effect for Virchow-2: pseudo-labels alone lifted F1 from 0.740 to 0.804 and reduced FP/slide from 5.64 to 3.80, while subsequent benign curation raised F1 to 0.832 and dropped FP/slide to 2.51 (Table S7). Data volume accounted for 70% of the F1 gain but only 59% of the FP reduction, confirming that curation contributed disproportionately to specificity, with a total gain of 12.4% relative F1 improvement and 55.5% FP reduction. Critically, precision improved from 0.678 to 0.828, while sensitivity simultaneously increased, demonstrating that the SSL expansion did not merely increase recall at the cost of more false alarms. This simultaneous improvement in both precision and sensitivity is a hallmark of the enriched benign class representation, which is analysed in detail in (Section 3.3). For UNI, whose baseline already handled benign mimics well, the SSL pipeline yielded a negligible F1 change (0.826 to 0.829); this asymmetry supports the interpretation that curation primarily addresses backbone-specific blind spots rather than a generic data-volume effect.

#### 3.2.2 Per-class detection performance

Beyond PNI, our framework simultaneously detects tumour and nerve tissue. For tumour detection, both pathology-pretrained models achieved near-perfect performance: Virchow-2 SSL reached F1 = 0.952 (precision = 0.955, 2.80 FP/slide) and UNI SSL reached F1 = 0.954 (precision = 0.970, 2.09 FP/slide), whereas EfficientNet-B3 and ConvNeXt-2 achieved high recall (0.98) but substantially lower precision (0.20 and 0.15), indicating extensive over-prediction of tumour regions (Fig. 3B). Nerve detection proved the most challenging task across all architectures. Among the foundation models, the strongest nerve F1 scores were obtained under baseline training (UNI 0.836, Virchow-2 0.832); under SSL, UNI retained the higher score (F1 = 0.810, precision = 0.896, 0.98 FP/slide), whereas Virchow-2 showed a modest drop (F1 = 0.756), suggesting that SSL expansion marginally reduced nerve specificity while improving PNI discrimination. Both CNN models showed high recall but very low precision for nerve detection, substantially over-detecting nerve-like structures. (EfficientNet-B3 SSL and ConvNeXt-2 SSL produced 22.9 and 40.4 FP per slide respectively; Table S5). These results confirm that pathology-pretrained foundation models domain-specific pretraining provides generalizable histological feature representations across all tissue classes, not only PNI.

#### 3.2.3 Threshold sensitivity analysis

To assess the robustness of each model across operating points, we evaluated PNI detection performance at confidence thresholds ranging from 50% to 90% (Fig. 3C). Virchow-2 SSL maintained recall above 0.83 from 50% through 70% and peaked at t = 70 (F1 = 0.832); UNI peaked earlier at t = 60 (F1 = 0.829), reflecting its more confident calibration. In contrast, EfficientNet-B3 and ConvNeXt-2 exhibited sharp recall degradation beyond 70%, with both models falling below 0.05 sensitivity at 90%, indicating that their predictions are poorly calibrated, few patches receive high-confidence PNI scores.

#### 3.2.4 Per-slide performance variability

Per-slide F1 score analysis (Fig. 3E) revealed that Virchow-2 with SSL achieved the highest median F1 score with a compact IQR among Virchow-2 conditions, indicating that the SSL pipeline not only raised average performance but also reduced per-slide variability. The stabilising effect was most pronounced in pancreatic tissue, where the BL produced the widest per-slide spread. UNI maintained consistently narrow distributions under both BL and SSL conditions, reflecting its already-strong performance across organs. Both CNN architectures showed wider variability and lower median F1 scores under all training conditions. Average Precision comparison (Fig. 3F) confirmed the consistency of the SSL benefit across all four architectures.

### 3.3 Benign class enrichment drives false positive reduction

**Figure 4:**
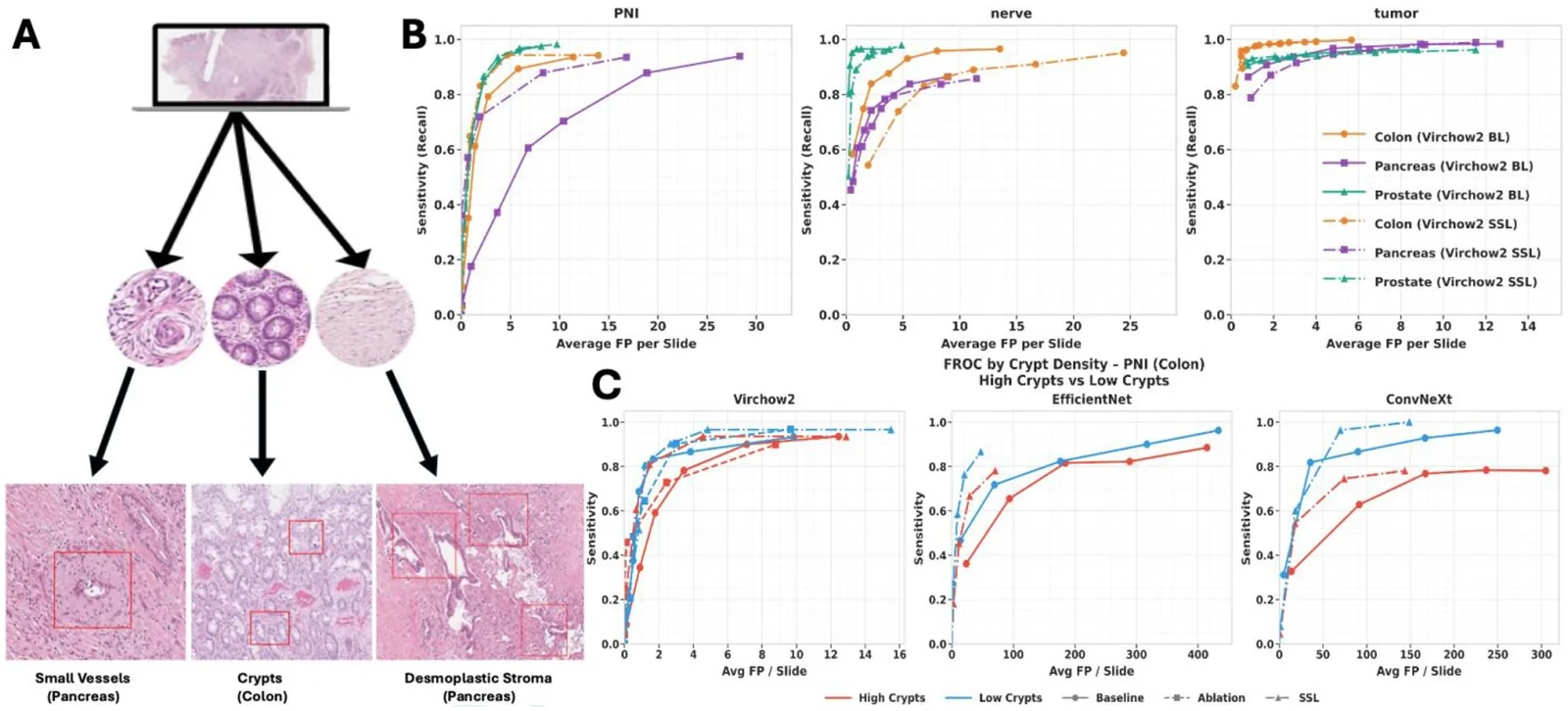
Organ-level error analysis. (A) Representative tissue WSI where non-neoplastic morphologies generate false positives: small vessels (left), crypts (centre), desmoplastic stroma (right). (B) FROC curves of baseline vs SSL for Virchow-2 to predict PNI (left), Nerve (centre), and Tumour (right) detection in organ type colon (orange), pancreas (purple), prostate (green). (C) FROC curves stratified by crypt density in colon tissue for Virchow-2 (left), EfficientNet-B3 (centre), ConvNeXt-2 (right), comparing high (red) vs. low (blue) crypt density under baseline (circle), ablation (square), and SSL (triangle) approaches.

Models trained on the baseline dataset (n = 262) produced systematic false positive predictions for specific non-neoplastic morphologies (Fig. 4A). Desmoplastic stroma in pancreatic tissue, crypts in colorectal tissue, and small blood vessels were frequently misclassified as PNI or nerve tissue due to their morphological similarity. These errors persisted even at high confidence thresholds because the initial training data did not adequately represent these structures in the benign class. The curation step described in (Section 2.4) addressed this gap, and the expanded benign class, enriched with these morphologies, transformed the pipeline from a data expansion strategy into a targeted mechanism for reducing false positives. Quantitatively, benign-class annotation coverage more than doubled, with the impact most visible in pancreatic tissue where desmoplastic stroma cases saw FP counts drop from over 25 to single digits after curation.

#### 3.3.1 Per-organ evidence for benign class impact

The FROC curves stratified by organ type in Fig. 4B and Fig. S1 demonstrate considerable variation in detection performance across tissues. Pancreatic adenocarcinoma showed the largest gain for Virchow-2, where the baseline failed most severely (F1 = 0.597; 10.4 FP/slide) due to desmoplastic stroma being systematically misclassified as PNI. After SSL, F1 rose to 0.781 and FP/slide fell by 82% to 1.87. Under baseline training, EfficentNet-B3 and ConvNeXt-2 produced over 600 FP per slide at comparable sensitivity levels. Both CNN baselines struggled considerably in this tissue type, requiring over 100 FP/slide to reach comparable sensitivity even after SSL expansion. Prostate, where Virchow-2’s baseline was already strong (F1 = 0.866), plausibly reflecting prostate’s 6.5% share of Virchow-2’s pretraining corpus, gained the least, with F1 rising only to 0.875 and FP/slide unchanged at 3.7. Colon showed a moderate gain (F1 0.760 to 0.817; FP/slide 2.73 to 1.93), consistent with partial curation of crypt-mimic patterns. Across all three organs, Virchow-2 maintained a clear advantage over both CNN baselines, reaching greater than 0.90 sensitivity at approximately 20 FP/slide. For UNI, whose baseline was already strong across all organs (Colon F1 = 0.841, Pancreas F1 = 0.778, Prostate F1 = 0.857), SSL provided minimal per-organ improvement, with FP/slide reductions concentrated in pancreas (2.93 to 0.40) but without meaningful F1 gains. SSL benefit in Virchow-2 was seen occurred in prostate tissue, where benign mimics are more morphologically diverse and subtle (making the enrichment of the benign class particularly impactful (Section 4.3)). Stratifying colon performance by crypt density (Fig. 4C) confirmed crypts as a complementary source of FP. Slides with high crypt density showed the largest baseline-to-SSL improvement across all architectures, while low-density slides showed smaller deltas.

## 4 Discussion

### 4.1 The foundation model advantage

Foundation models substantially outperform CNNs across every training condition, establishing the benefit of pathology specific domain pretraining as a prerequisite rather than a tuneable design choice. EfficientNet and ConvNeXt, initialised on natural images (ImageNet), struggle to differentiate the texture of a nerve fibre from stromal connective tissue, patterns that rarely occur in natural images. UNI and Virchow-2 showed distinct baseline profiles. UNI was stronger out of the box (F1 = 0.826 vs 0.740) despite roughly half the parameter count of Virchow-2 (307M vs 632M) and a substantially smaller pretraining corpus (∼100K vs 3.1M WSIs); this advantage therefore cannot be attributed to scale alone. We attribute UNI’s stronger baseline to its training corpus containing greater representation of inflammatory and reactive tissue structures that closely mimic PNI on H&E (Klöppel and Adsay 2009, Chen *et al.* 2024). Virchow-2’s larger pretraining corpus (3.1M vs 100K WSIs) ultimately produced the highest post-SSL F1 (0.832). This pattern suggests that ImageNet-pretrained models lack the representational vocabulary to distinguish genuine nerve fibres from visually similar stromal structures, a distinction that pathology-specific pretraining provides; the model-specific error profiles further indicate that pretraining-data composition, not just volume, determines where downstream curation pays off.

### 4.2 Effectiveness of confidence-based semi-supervised learning

Confidence-thresholded pseudo-labelling is well established in semi-supervised learning (Sohn *et al.* 2020). Our contribution lies not in the pseudo-labelling mechanism itself but in its application as a targeted data curation strategy for PNI detection, where the primary bottleneck is the inadequate representation of morphologically ambiguous benign structures rather than annotation volume. This curation strategy provided substantial and consistent improvements across all four architectures, with the most pronounced gains observed for Virchow-2 (12.4% F1 improvement, 55.5% FP reduction). The consistency of the SSL benefit across architecturally diverse models, from lightweight CNNs to large-scale vision transformers, suggests that the improvement stems from the quality of the expanded training data rather than architecture-specific interactions. Our framework’s F1 of 0.832 compares favourably with the top PAIP (2021) challenge score of 0.597 (PAIP 2021), though direct comparison is limited by differences in evaluation paradigm (region-level detection vs pixel-level segmentation), test set composition, and training data availability (352 WSIs with SSL vs. 150 WSIs for challenge participants). Published challenges methods include a U-Net boundary dilation method with F1 of 0.275 (Park *et al.* 2022), both relying on ImageNet-CNNs without semi-supervised data expansion.

### 4.3 The role of benign class representation

The primary mechanism through which SSL improves PNI detection is targeted enrichment of the benign class with morphologies that systematically generate FP in the baseline. The per-organ pattern in (Section 3.3.1), largest gain in pancreas, minimal gain in prostate, flat UNI response, is direct evidence that curation corrects organ-specific benign-class gaps rather than acting as generic data augmentation and can be explained by the distinct histomorphological characteristics of each tissue type. The most modest SSL improvement was observed in prostate tissue, where Virchow-2’s baseline was already strong (F1 = 0.866), plausibly because prostatic glandular tissue is well-represented in Virchow-2’s pretraining corpus; nonetheless, the known diagnostic complexity of prostate PNI, with inter-observer kappa values of only 0.67–0.75 arising from stromal bundles and smooth muscle mimicking nerve tissue (Trpkov 2018, Egevad *et al.* 2021), underscores the clinical value of automated detection even where model performance is high. Colon showed a moderate SSL gain, consistent with partial curation of crypt-like benign patterns that mimic nerve morphology in H&E sections (Liebig *et al*. 2009b, Wang *et al.* 2024). The under-reporting of PNI in colorectal cancer, with detection rates as low as 16% in routine pathology reports compared to 22% on expert re-review (Liebig *et al*. 2009b), highlights the clinical need for automated detection tools across all organ types. The largest SSL benefit was observed in pancreatic tissue, where the baseline failed most severely, and this can be directly attributed to the nature of desmoplastic stroma in pancreas. PDAC is characterised by extensive desmoplasia, where fibrotic stromal tissue rich in extracellular matrix components may represent as much as 90% of the overall tumour mass (Wang *et al.* 2016, Sperb *et al.* 2020). PNI occurs in 70–100% of PDAC cases (Selvaggi *et al.* 2022), meaning nerve tissue and desmoplastic stroma co-occur almost ubiquitously, and curating these regions into the benign class directly addressed the dominant baseline failure mode, reducing FP/slide from 10.4 to 1.87. This demonstrates that the challenge in pancreatic tissue was not data scarcity but the absence of representative desmoplastic stroma in the benign class. The contrasting SSL response of UNI is informative: UNI’s pretraining corpus contains greater representation of inflammatory and reactive tissue morphologies that closely mimic PNI on H&E, likely pre-equipping it to handle many of the benign structures that Virchow-2 had to learn through curation (Klöppel and Adsay 2009, Chen *et al.* 2024). This may explain why UNI achieved a strong baseline with minimal SSL benefit, while Virchow-2 required targeted benign-class enrichment to reach comparable performance, arguing for pretraining-aware curation budgets. While this study demonstrates strong performance within the PAIP dataset, external validation on independent multi-institutional cohorts is needed to confirm generalisability across centres. Future work will focus on expanding validation to additional organ types and prospective clinical evaluation.

## 5 Conclusion

This work presents a framework for automated PNI detection that addresses the critical shortage of annotated pathological data through three complementary strategies. First, two pathology-pretrained foundation models, Virchow-2 and UNI, substantially outperformed ImageNet-pretrained CNN baselines, confirming that domain-specific foundation models provide a critical advantage for detecting subtle histopathological features. Second, our confidence-thresholding SSL strategy (*P* > .9) expanded the training set from 262 to 352 WSIs while maintaining label quality through strict confidence filtering and human-in-the-loop curation, yielding a 12.4% relative F1 improvement and 55.5% FP reduction for Virchow-2. Third, per-organ analysis revealed that curation benefit scales inversely with baseline performance, with the largest gain in pancreas, where desmoplastic stroma systematically mimics PNI, and the most modest gain in prostate, where Virchow-2’s baseline was already strong. This study provides a framework to deliver high quality pathology annotations masks that are critical for assessment, segmentation, and interpretation of single cell spatial “omics data.”

## Supplementary Material

btag461_Supplementary_Data

## Data Availability

The whole slide images and annotation masks used in this study were obtained from the PAIP 2021 Challenge: Perineural Invasion in Multiple Organ Cancer (Colon, Prostate and Pancreatobiliary Tract) dataset. These data are not publicly available without restriction but can be accessed through the PAIP challenge portal upon completion and acceptance of the required Data Use and Confidentiality Agreement.
