# Supplementary material for: Semi-supervised learning for automated perineural invasion detection in multi-organ H&E whole slide images
Source: Bioinformatics. 2026 Aug 21;42(Suppl 2):btag461. doi: 10.1093/bioinformatics/btag461 (PMC13501286; doi:10.1093/bioinformatics/btag461)

Supplementary Data for "*Semi-Supervised Learning for Automated Perineural Invasion Detection in Multi-Organ H&E Whole Slide Images*"

A Alkhan[1*], M Lynch[1], M Lavelle[2], and AC Culhane[1]

[1]School of Medicine, Limerick Digital Cancer Research, and Health Research Institute, University of Limerick, Limerick, Co. Limerick, V94 T9PX, Ireland.

[2]Pathology Department, University Hospital Limerick, Limerick, Co. Limerick, V94 F858, Ireland.

*Corresponding author, email: ahmad.alkhan@ul.ie

Associate Editor: XXXXXXX

Received on XXXXX; revised on XXXXX; accepted on XXXXX

Supplementary

1. Hyperparameters, and Training strategy

All models were trained using PyTorch with automatic mixed precision (AMP) and a random seed of 42 for reproducibility. The two convolutional models, EfficientNet-B3 (12M parameters, pretrained on ImageNet-1k with RandAugment) and ConvNeXtV2-Base (89M parameters, pretrained on ImageNet-22k), were trained end-to-end with layer-wise learning rate decay (decay factor 0.65–0.80) and a backbone learning rate multiplier of 0.1 relative to the classifier head. Virchow-2 (ViT-H/14, 632M parameters) and UNI (ViT-L/16, 307M parameters) both had their pretrained backbones frozen throughout all training stages. A two-stage training protocol was used: Stage 1 trained multi-layer classification heads (Virchow-2: FC[2560→512→256→4]; UNI: FC[1024→512→256→4], both with GELU activations and LayerNorm) on all four classes (PNI, Tumor, Nerve, Benign). Stage 2 fine-tuned binary refinement classifiers per class group to filter Stage 1 false positives, using layer-wise learning rate decay (factor 0.75) to preserve the frozen pretrained representations. Learning rates for the classification heads ranged from 1×10^{-3} to 1×10^{-5}. All classifier heads were trained on 224×224-pixel patches extracted at two magnification levels (single level for Virchow2). Optimisation used AdamW with weight decay of 0.01–0.05, cosine annealing learning rate scheduling with linear warm-up (1–8 epochs), and gradient clipping at a norm of 1.0. Learning rates ranged from 1×10^{-3} (Virchow2 linear probe) to 1×10^{-5} (Virchow2 fine-tuning), with intermediate values of 2×10^{-4} and 5×10^{-5} for EfficientNet-B3 and ConvNeXtV2, respectively. Effective batch sizes of 32–96 were achieved through gradient accumulation. To handle the severe class imbalance inherent in PNI detection, training employed a class-balanced focal loss (γ=2.0, β=0.9999) with an additional false-negative penalty term for PNI (penalty weight=2.0–3.0), label smoothing (ε=0.05–0.15), and a progressive weighted random sampler that linearly increased class-balancing weight from 30% to 100% over training. Patch counts per class were capped at 10,000–20,000 to prevent majority-class dominance. Data augmentation included random horizontal and vertical flips, 90° rotations, affine transforms (shift±10%, scale±15%, rotation±15°), color jitter (brightness/contrast/saturation ±20%), H&E stain jitter (θ=0.05), Gaussian noise, coarse dropout, and elastic deformation; PNI patches received a more aggressive augmentation schedule given their scarcity. Early stopping was applied based on validation macro-F1 (or PNI-specific F1 for Stage 2 models) with a patience of 5–25 epochs. For the non-SSL (PAIP-only) experiments, the same architecture and training procedure were used but with training data restricted to the PAIP 2021 challenge and WISEPAIP datasets, excluding the additional Unlabelled data (n=90) used in the SSL pipeline.

To handle the multi-organ nature of the data, we trained a single encoder for all the organs, as PNI morphological features looks quite similar between the three organs. Using specific-organ encoders could be beneficial in case large dataset of the different organs are available. On the other hand, our approach ensures the encoders trains on many different examples of PNI from different organs. At inference stage, a different thresholds values were used to find the optimum threshold that gives the least false positives per slides. In addition, activation maps were also generated, which can help

visualise the model's attention when calling PNI patches (Figure 2B). This architecture allows the model to learn wide range of PNI morphologies from different organs.

2. Evaluation Metrics

Performance was assessed using region-level detection metrics. Since models produce 224×224 -pixel patch predictions, overlapping and adjacent patches were first merged using buffered geometric union (ε = 1.0 pixel) to consolidate multiple patches covering the same ground truth annotation into a single detection region. Detection matching employed a touch-based criterion: a merged prediction was counted as a True Positive (TP) if it spatially intersected any ground truth annotation, as a False Positive (FP) if it touched no ground truth, while undetected ground truth regions were counted as False Negatives (FN). This approach was preferred over (Intersection over Union) IoU-based matching due to the size mismatch between fixed-size patches and variable-extent annotations.

From these counts, the following metrics were computed:

Precision: The fraction of detections that are correct, calculated as TP / (TP + FP).

Recall (Sensitivity): The fraction of ground truth annotations that are detected, calculated as TP / (TP + FN).

F1-Score: The harmonic mean of Precision and Recall, calculated as 2 × (Precision × Recall) / (Precision + Recall).

Average Precision (AP): A threshold-independent summary computed using Visual object classes (VOC)-style all-point interpolation of the Precision-Recall curve, representing the area under the interpolated PR curve.

Precision, Recall, and F1-Score were computed both globally (aggregated across all 45 test slides) and per-slide to characterize performance variability. Free-Response ROC (FROC) curves were generated to assess clinical utility, plotting Sensitivity against the average number of False Positives per slide across confidence thresholds. This analysis reveals the trade-off between detection sensitivity and false alarm rate at varying operating points. Per-organ FROC analysis was conducted for Colon, Pancreas, and Prostate subsets (15 slides each) to evaluate tissue-specific performance variation.

3. Computational cost and training time

To assess the practical computational requirements of each architecture, we compared training time, inference time, and cloud computing costs across all three backbones (Tables S1–S3). EfficientNet-B3 and ConvNeXt-2 were trained locally on an NVIDIA A30 GPU, while Virchow-2 was trained on an AWS g5.2xlarge instance (NVIDIA A10G GPU, on-demand rate $1.30/hr). All models used early stopping based on validation loss, which terminated EfficientNet-B3 at 39/50 epochs and ConvNeXt-2 at 21/50 epochs for the SSL pipeline; Virchow-2 SSL was trained for a fixed 20 epochs. EfficientNet-B3 was the most efficient model, completing SSL training in 285 minutes (7.3 min/epoch) compared to 774 minutes for ConvNeXt-2 (36.9 min/epoch) and 1,084 minutes for Virchow-2 (54.2 min/epoch), reflecting the substantial difference in parameter count (11.1M vs. 88.2M vs. 632M; Table S1). Notably, ConvNeXt-2 baseline training required 1,307 minutes across its full 50 epochs, exceeding even Virchow-2 SSL training (1,084 minutes at 20 epochs), indicating that ConvNeXt-2's per-epoch cost is high relative to its detection performance. Virchow-2 training on the PAIP-only baseline required only 416 minutes (26 epochs), as the frozen backbone meant that only the linear classification head was updated; the SSL pipeline's longer training time reflects the larger expanded dataset (67,000 vs. 19,697 training samples). At inference, Virchow-2 required a mean of 386 seconds per slide compared to 223 seconds for EfficientNet-B3 and 258 seconds for ConvNeXt-2 (Table S2), with colon slides consistently requiring the longest inference time across all models due to larger tissue area. The total cloud computing cost for all Virchow-2 experiments, including both training pipelines and inference on the 45-slide test set, was $44.97 (Table S3), demonstrating that foundation model-based PNI detection is feasible within modest computational budgets despite the model's 632M parameters.

Table S1. Training time comparison across model architectures. EfficientNet-B3 and ConvNeXt-2 were trained on a local NVIDIA A30 GPU; Virchow-2 was trained on an AWS g5.2xlarge instance (NVIDIA A10G).

**Model**	**Pipeline**	**Parameters**	**Training Samples**	**Epochs**	**Training Time (min)**	**Time/Epoch (min)**	**EC2 Cost ($)**
EfficientNet-B3	SSL	11.1M	80,782	39/50	285	7.3	N/A (local)
EfficientNet-B3	Baseline	11.1M	53,623	50/50	254	5.1	N/A (local)
ConvNeXt-2	SSL	88.2M	80,782	21/50	774	36.9	N/A (local)
ConvNeXt-2	Baseline	88.2M	53,623	50/50	1,307	26.1	N/A (local)
Virchow-2	SSL	632.0M	~67,000	20/20	1,084	54.2	$23.49
Virchow-2	Baseline	632.0M	19,697	26/50	416	16.0	$9.01

Table S2. Inference time comparison on the holdout test set (n=45 WSIs; 15 per organ). All models were evaluated on the same hardware used for training.

**Model**	**Mean Time/Slide (sec)**	**Total 45 Slides (min)**	**Colon (sec)**	**Pancreas (sec)**	**Prostate (sec)**
EfficientNet-B3	223.4	167.5	283.2	206.2	180.8
ConvNeXt-2	257.6	193.2	316.6	240.7	215.5
Virchow-2 (SSL)	386.4	289.8	466.7	363.7	329.0
Virchow-2 (Baseline)	381.3	285.9	400.4	383.5	359.9

Table S3. AWS EC2 cost breakdown for all Virchow-2 experiments (g5.2xlarge instance, $1.30/hr on-demand).

**Task**	**Duration (hrs)**	**Cost ($)**
SSL Training	18.07	$23.49
SSL Inference	4.83	$6.28
Baseline Training	6.93	$9.01
Baseline Inference	4.76	$6.19
**Grand Total**	**34.59**	**$44.97**

4. Statistical Analysis

To assess the significance of performance differences between the SSL and non-SSL training approaches, we employed three complementary statistical methods on the aggregated detection counts (TP, FP, FN) across all 45 test slides. For each model (Virchow, EfficientNet-B3, ConvNeXtV2-B) and class (PNI, tumor, nerve), we selected the probability threshold that maximized F1 score independently for each approach, reflecting the operational setting where each model would be deployed at its own optimal threshold. Precision, being a proportion (TP/(TP+FP)), was evaluated using Wilson score 95% confidence intervals and two-proportion z-tests comparing the SSL and non-SSL rates. For F1 score, which is a non-linear function of precision and recall, we used a parametric bootstrap procedure (10,000 resamples): true positive counts were resampled from a Binomial distribution parameterized by the observed precision rate, and both precision and recall were recomputed from the resampled TP to preserve their natural correlation through the shared detection count. P-values for F1 were obtained via a permutation-style bootstrap under the null hypothesis, where both approaches were resampled from a pooled precision rate. Average Precision (AP) was computed using VOC-style all-point interpolation

across the precision-recall curve, with bootstrap confidence intervals and p-values obtained by resampling TP at each threshold and recomputing the full curve. All tests were two-sided, and statistical significance was set at $p < 0.05$.

Table S4. Statistical Comparison of Baseline vs SSL Detection Performance

Model	Class	Metric	SSL Value	SSL 95% CI	SSL Thresh	Baseline Value	Baseline 95% CI	Baseline Thresh	Diff	P Value
Virchow2	PNI	Precision	0.828	[0.797, 0.855]	70%	0.678	[0.645, 0.710]	70%	+0.150	p<0.0001
Virchow2	PNI	F1	0.832	[0.802, 0.859]	70%	0.740	[0.704, 0.775]	70%	+0.092	p=0.1297
Virchow2	tumor	Precision	0.955	[0.947, 0.962]	75%	0.976	[0.969, 0.981]	85%	-0.021	p<0.0001
Virchow2	tumor	F1	0.952	[0.945, 0.960]	75%	0.956	[0.950, 0.961]	85%	-0.003	p=0.9986
Virchow2	nerve	Precision	0.723	[0.684, 0.759]	80%	0.859	[0.824, 0.888]	80%	-0.136	p<0.0001
Virchow2	nerve	F1	0.756	[0.716, 0.795]	80%	0.832	[0.800, 0.861]	80%	-0.075	p=0.3042
UNI	PNI	Precision	0.788	[0.756, 0.816]	60%	0.843	[0.813, 0.869]	70%	-0.055	p=0.0088
UNI	PNI	F1	0.829	[0.797, 0.859]	60%	0.826	[0.798, 0.853]	70%	+0.003	p=0.9984
UNI	tumor	Precision	0.970	[0.964, 0.976]	70%	0.972	[0.966, 0.977]	85%	-0.002	p=0.6386
UNI	tumor	F1	0.954	[0.948, 0.960]	70%	0.960	[0.955, 0.966]	85%	-0.006	p=0.3235
UNI	nerve	Precision	0.896	[0.864, 0.922]	75%	0.885	[0.853, 0.912]	85%	+0.011	p=0.6095
UNI	nerve	F1	0.810	[0.785, 0.836]	75%	0.836	[0.807, 0.863]	85%	-0.026	p=0.7022
EfficientNet	PNI	Precision	0.443	[0.411, 0.475]	70%	0.296	[0.270, 0.324]	90%	+0.146	p<0.0001
EfficientNet	PNI	F1	0.505	[0.469, 0.541]	70%	0.360	[0.329, 0.393]	90%	+0.144	p<0.0001
EfficientNet	tumor	Precision	0.423	[0.411, 0.435]	90%	0.364	[0.355, 0.374]	90%	+0.058	p<0.0001
EfficientNet	tumor	F1	0.557	[0.541, 0.572]	90%	0.512	[0.498, 0.526]	90%	+0.045	p=0.1619
EfficientNet	nerve	Precision	0.200	[0.179, 0.222]	80%	0.108	[0.098, 0.120]	90%	+0.091	p<0.0001
EfficientNet	nerve	F1	0.284	[0.253, 0.314]	80%	0.186	[0.166, 0.205]	90%	+0.098	p=0.0007
ConvNeXt	PNI	Precision	0.265	[0.245, 0.287]	70%	0.382	[0.344, 0.421]	90%	-0.116	p<0.0001
ConvNeXt	PNI	F1	0.383	[0.352, 0.414]	70%	0.339	[0.304, 0.374]	90%	+0.044	p=1.0000
ConvNeXt	tumor	Precision	0.370	[0.355, 0.385]	90%	0.392	[0.380, 0.404]	90%	-0.022	p=0.0226
ConvNeXt	tumor	F1	0.452	[0.433, 0.471]	90%	0.523	[0.507, 0.538]	90%	-0.071	p=0.0101
ConvNeXt	nerve	Precision	0.147	[0.132, 0.162]	80%	0.122	[0.109, 0.135]	90%	+0.025	p=0.0122
ConvNeXt	nerve	F1	0.239	[0.215, 0.264]	80%	0.205	[0.184, 0.226]	90%	+0.034	p=0.0639

Table S5. Per-class summary results for the SSL approach

Class	Model	Organ	Threshold	TP	FP	FN	Precision	Recall	F1	Avg_FP_per_Slide
PNI	Virchow2	Colon	50%	130	209	8	0.3835	0.942	0.5451	13.93
PNI	Virchow2	Colon	60%	132	70	8	0.6535	0.9429	0.7719	4.67
PNI	Virchow2	Colon	70%	118	29	24	0.8027	0.831	0.8166	1.93
PNI	Virchow2	Colon	75%	96	13	52	0.8807	0.6486	0.7471	0.87
PNI	Virchow2	Colon	80%	45	6	101	0.8824	0.3082	0.4569	0.4
PNI	Virchow2	Colon	85%	4	0	146	1	0.0267	0.0519	0
PNI	Virchow2	Colon	90%	0	0	151	0	0	0	0
PNI	Virchow2	Pancreas	50%	216	252	15	0.4615	0.9351	0.618	16.8
PNI	Virchow2	Pancreas	60%	203	125	28	0.6189	0.8788	0.7263	8.33
PNI	Virchow2	Pancreas	70%	166	28	65	0.8557	0.7186	0.7812	1.87

PNI	Virchow2	Pancreas	75%	137	10	103	0.932	0.5708	0.708	0.67
PNI	Virchow2	Pancreas	80%	88	0	156	1	0.3607	0.5301	0
PNI	Virchow2	Pancreas	85%	16	0	222	1	0.0672	0.126	0
PNI	Virchow2	Pancreas	90%	0	0	242	0	0	0	0
PNI	Virchow2	Prostate	50%	271	146	5	0.6499	0.9819	0.7821	9.73
PNI	Virchow2	Prostate	60%	270	89	9	0.7521	0.9677	0.8464	5.93
PNI	Virchow2	Prostate	70%	259	56	18	0.8222	0.935	0.875	3.73
PNI	Virchow2	Prostate	75%	239	34	37	0.8755	0.8659	0.8707	2.27
PNI	Virchow2	Prostate	80%	182	16	99	0.9192	0.6477	0.7599	1.07
PNI	Virchow2	Prostate	85%	46	0	243	1	0.1592	0.2746	0
PNI	Virchow2	Prostate	90%	0	0	291	0	0	0	0
PNI	Virchow2	All	50%	617	607	28	0.5041	0.9566	0.6602	13.49
PNI	Virchow2	All	60%	605	284	45	0.6805	0.9308	0.7862	6.31
PNI	Virchow2	All	70%	543	113	107	0.8277	0.8354	0.8315	2.51
PNI	Virchow2	All	75%	472	57	192	0.8922	0.7108	0.7913	1.27
PNI	Virchow2	All	80%	315	22	356	0.9347	0.4694	0.625	0.49
PNI	Virchow2	All	85%	66	0	611	1	0.0975	0.1777	0
PNI	Virchow2	All	90%	0	0	684	0	0	0	0
PNI	UNI	Colon	50%	126	205	12	0.3807	0.913	0.5373	13.67
PNI	UNI	Colon	60%	125	48	17	0.7225	0.8803	0.7937	3.2
PNI	UNI	Colon	70%	89	9	53	0.9082	0.6268	0.7417	0.6
PNI	UNI	Colon	75%	59	2	88	0.9672	0.4014	0.5673	0.13
PNI	UNI	Colon	80%	23	0	127	1	0.1533	0.2659	0
PNI	UNI	Colon	85%	2	0	149	1	0.0132	0.0261	0
PNI	UNI	Colon	90%	0	0	151	0	0	0	0
PNI	UNI	Pancreas	50%	208	167	22	0.5547	0.9043	0.6876	11.13
PNI	UNI	Pancreas	60%	191	50	41	0.7925	0.8233	0.8076	3.33
PNI	UNI	Pancreas	70%	152	6	93	0.962	0.6204	0.7543	0.4
PNI	UNI	Pancreas	75%	110	2	135	0.9821	0.449	0.6162	0.13
PNI	UNI	Pancreas	80%	53	0	188	1	0.2199	0.3605	0
PNI	UNI	Pancreas	85%	5	0	236	1	0.0207	0.0407	0
PNI	UNI	Pancreas	90%	0	0	242	0	0	0	0
PNI	UNI	Prostate	50%	263	101	13	0.7225	0.9529	0.8219	6.73
PNI	UNI	Prostate	60%	255	56	24	0.8199	0.914	0.8644	3.73
PNI	UNI	Prostate	70%	226	34	56	0.8692	0.8014	0.8339	2.27
PNI	UNI	Prostate	75%	172	20	112	0.8958	0.6056	0.7227	1.33
PNI	UNI	Prostate	80%	76	8	214	0.9048	0.2621	0.4064	0.53
PNI	UNI	Prostate	85%	8	0	282	1	0.0276	0.0537	0
PNI	UNI	Prostate	90%	0	0	291	0	0	0	0
PNI	UNI	All	50%	597	473	47	0.5579	0.927	0.6966	10.51
PNI	UNI	All	60%	571	154	82	0.7876	0.8744	0.8287	3.42
PNI	UNI	All	70%	467	49	202	0.905	0.6981	0.7882	1.09

PNI	UNI	All	75%	341	24	335	0.9342	0.5044	0.6551	0.53
PNI	UNI	All	80%	152	8	529	0.95	0.2232	0.3615	0.18
PNI	UNI	All	85%	15	0	667	1	0.022	0.043	0
PNI	UNI	All	90%	0	0	684	0	0	0	0
PNI	EfficientNet	Colon	50%	108	915	27	0.1056	0.8	0.1865	61
PNI	EfficientNet	Colon	60%	107	382	48	0.2188	0.6903	0.3323	25.47
PNI	EfficientNet	Colon	70%	83	160	88	0.3416	0.4854	0.401	10.67
PNI	EfficientNet	Colon	80%	42	39	165	0.5185	0.2029	0.2917	2.6
PNI	EfficientNet	Colon	90%	0	1	236	0	0	0	0.07
PNI	EfficientNet	Pancreas	50%	160	1082	28	0.1288	0.8511	0.2238	72.13
PNI	EfficientNet	Pancreas	60%	155	359	49	0.3016	0.7598	0.4318	23.93
PNI	EfficientNet	Pancreas	70%	153	139	90	0.524	0.6296	0.572	9.27
PNI	EfficientNet	Pancreas	80%	93	46	203	0.6691	0.3142	0.4276	3.07
PNI	EfficientNet	Pancreas	90%	6	1	314	0.8571	0.0187	0.0367	0.07
PNI	EfficientNet	Prostate	50%	213	661	56	0.2437	0.7918	0.3727	44.07
PNI	EfficientNet	Prostate	60%	201	383	77	0.3442	0.723	0.4664	25.53
PNI	EfficientNet	Prostate	70%	177	221	113	0.4447	0.6103	0.5145	14.73
PNI	EfficientNet	Prostate	80%	126	97	172	0.565	0.4228	0.4837	6.47
PNI	EfficientNet	Prostate	90%	19	10	300	0.6552	0.0596	0.1092	0.67
PNI	EfficientNet	All	50%	481	2658	111	0.1532	0.8125	0.2578	59.07
PNI	EfficientNet	All	60%	463	1124	174	0.2917	0.7268	0.4164	24.98
PNI	EfficientNet	All	70%	413	520	291	0.4427	0.5866	0.5046	11.56
PNI	EfficientNet	All	80%	261	182	540	0.5892	0.3258	0.4196	4.04
PNI	EfficientNet	All	90%	25	12	850	0.6757	0.0286	0.0548	0.27
PNI	ConvNeXt	Colon	50%	102	2184	21	0.0446	0.8293	0.0847	145.6
PNI	ConvNeXt	Colon	60%	106	1087	28	0.0889	0.791	0.1598	72.47
PNI	ConvNeXt	Colon	70%	90	273	72	0.2479	0.5556	0.3429	18.2
PNI	ConvNeXt	Colon	80%	12	16	218	0.4286	0.0522	0.093	1.07
PNI	ConvNeXt	Colon	90%	0	0	236	0	0	0	0
PNI	ConvNeXt	Pancreas	50%	140	3744	16	0.036	0.8974	0.0693	249.6
PNI	ConvNeXt	Pancreas	60%	150	1980	26	0.0704	0.8523	0.1301	132
PNI	ConvNeXt	Pancreas	70%	162	639	52	0.2022	0.757	0.3192	42.6
PNI	ConvNeXt	Pancreas	80%	42	34	266	0.5526	0.1364	0.2188	2.27
PNI	ConvNeXt	Pancreas	90%	0	0	323	0	0	0	0
PNI	ConvNeXt	Prostate	50%	228	885	25	0.2049	0.9012	0.3338	59
PNI	ConvNeXt	Prostate	60%	218	603	43	0.2655	0.8352	0.403	40.2
PNI	ConvNeXt	Prostate	70%	196	329	78	0.3733	0.7153	0.4906	21.93
PNI	ConvNeXt	Prostate	80%	67	41	238	0.6204	0.2197	0.3245	2.73
PNI	ConvNeXt	Prostate	90%	0	0	323	0	0	0	0
PNI	ConvNeXt	All	50%	470	6813	62	0.0645	0.8835	0.1203	151.4
PNI	ConvNeXt	All	60%	474	3670	97	0.1144	0.8301	0.2011	81.56
PNI	ConvNeXt	All	70%	448	1241	202	0.2652	0.6892	0.3831	27.58

PNI	ConvNeXt	All	80%	121	91	722	0.5708	0.1435	0.2294	2.02
PNI	ConvNeXt	All	90%	0	0	882	0	0	0	0
tumor	Virchow2	Colon	50%	687	52	7	0.9296	0.9899	0.9588	3.47
tumor	Virchow2	Colon	60%	844	34	14	0.9613	0.9837	0.9724	2.27
tumor	Virchow2	Colon	70%	987	17	25	0.9831	0.9753	0.9792	1.13
tumor	Virchow2	Colon	75%	1024	11	40	0.9894	0.9624	0.9757	0.73
tumor	Virchow2	Colon	80%	996	7	65	0.993	0.9387	0.9651	0.47
tumor	Virchow2	Colon	85%	941	8	110	0.9916	0.8953	0.941	0.53
tumor	Virchow2	Colon	90%	844	3	173	0.9965	0.8299	0.9056	0.2
tumor	Virchow2	Pancreas	50%	620	173	7	0.7818	0.9888	0.8732	11.53
tumor	Virchow2	Pancreas	60%	707	137	14	0.8377	0.9806	0.9035	9.13
tumor	Virchow2	Pancreas	70%	817	98	34	0.8929	0.96	0.9253	6.53
tumor	Virchow2	Pancreas	75%	852	72	49	0.9221	0.9456	0.9337	4.8
tumor	Virchow2	Pancreas	80%	830	46	77	0.9475	0.9151	0.931	3.07
tumor	Virchow2	Pancreas	85%	818	28	122	0.9669	0.8702	0.916	1.87
tumor	Virchow2	Pancreas	90%	737	14	199	0.9814	0.7874	0.8737	0.93
tumor	Virchow2	Prostate	50%	628	173	25	0.784	0.9617	0.8638	11.53
tumor	Virchow2	Prostate	60%	647	102	33	0.8638	0.9515	0.9055	6.8
tumor	Virchow2	Prostate	70%	718	61	46	0.9217	0.9398	0.9307	4.07
tumor	Virchow2	Prostate	75%	802	43	53	0.9491	0.938	0.9435	2.87
tumor	Virchow2	Prostate	80%	898	31	59	0.9666	0.9383	0.9523	2.07
tumor	Virchow2	Prostate	85%	1068	15	80	0.9861	0.9303	0.9574	1
tumor	Virchow2	Prostate	90%	1372	11	120	0.992	0.9196	0.9544	0.73
tumor	Virchow2	All	50%	1935	398	39	0.8294	0.9802	0.8985	8.84
tumor	Virchow2	All	60%	2198	273	61	0.8895	0.973	0.9294	6.07
tumor	Virchow2	All	70%	2522	176	105	0.9348	0.96	0.9472	3.91
tumor	Virchow2	All	75%	2678	126	142	0.9551	0.9496	0.9523	2.8
tumor	Virchow2	All	80%	2724	84	201	0.9701	0.9313	0.9503	1.87
tumor	Virchow2	All	85%	2827	51	312	0.9823	0.9006	0.9397	1.13
tumor	Virchow2	All	90%	2953	28	492	0.9906	0.8572	0.9191	0.62
tumor	UNI	Colon	50%	808	54	22	0.9374	0.9735	0.9551	3.6
tumor	UNI	Colon	60%	981	23	41	0.9771	0.9599	0.9684	1.53
tumor	UNI	Colon	70%	1076	8	88	0.9926	0.9244	0.9573	0.53
tumor	UNI	Colon	75%	999	6	143	0.994	0.8748	0.9306	0.4
tumor	UNI	Colon	80%	835	1	207	0.9988	0.8013	0.8892	0.07
tumor	UNI	Colon	85%	583	1	250	0.9983	0.6999	0.8229	0.07
tumor	UNI	Colon	90%	277	0	310	1	0.4719	0.6412	0
tumor	UNI	Pancreas	50%	641	147	6	0.8135	0.9907	0.8934	9.8
tumor	UNI	Pancreas	60%	846	107	19	0.8877	0.978	0.9307	7.13
tumor	UNI	Pancreas	70%	1001	53	63	0.9497	0.9408	0.9452	3.53
tumor	UNI	Pancreas	75%	989	28	114	0.9725	0.8966	0.933	1.87
tumor	UNI	Pancreas	80%	829	11	198	0.9869	0.8072	0.8881	0.73

tumor	UNI	Pancreas	85%	549	6	283	0.9892	0.6599	0.7916	0.4
tumor	UNI	Pancreas	90%	225	0	385	1	0.3689	0.5389	0
tumor	UNI	Prostate	50%	652	145	28	0.8181	0.9588	0.8829	9.67
tumor	UNI	Prostate	60%	753	86	35	0.8975	0.9556	0.9256	5.73
tumor	UNI	Prostate	70%	977	33	49	0.9673	0.9522	0.9597	2.2
tumor	UNI	Prostate	75%	1062	17	64	0.9842	0.9432	0.9633	1.13
tumor	UNI	Prostate	80%	1225	3	76	0.9976	0.9416	0.9688	0.2
tumor	UNI	Prostate	85%	1346	3	109	0.9978	0.9251	0.9601	0.2
tumor	UNI	Prostate	90%	1362	0	173	1	0.8873	0.9403	0
tumor	UNI	All	50%	2101	346	56	0.8586	0.974	0.9127	7.69
tumor	UNI	All	60%	2580	216	95	0.9227	0.9645	0.9432	4.8
tumor	UNI	All	70%	3054	94	200	0.9701	0.9385	0.9541	2.09
tumor	UNI	All	75%	3050	51	321	0.9836	0.9048	0.9425	1.13
tumor	UNI	All	80%	2889	15	481	0.9948	0.8573	0.9209	0.33
tumor	UNI	All	85%	2478	10	642	0.996	0.7942	0.8837	0.22
tumor	UNI	All	90%	1864	0	868	1	0.6823	0.8111	0
tumor	EfficientNet	All	50%	928	14869	6	0.0587	0.9936	0.1109	330.42
tumor	EfficientNet	All	60%	1708	12764	27	0.118	0.9844	0.2108	283.64
tumor	EfficientNet	All	70%	2868	11169	65	0.2043	0.9778	0.338	248.2
tumor	EfficientNet	All	80%	4061	8661	209	0.3192	0.9511	0.478	192.47
tumor	EfficientNet	All	90%	2914	3978	659	0.4228	0.8156	0.5569	88.4
tumor	ConvNeXt	All	50%	733	11709	6	0.0589	0.9919	0.1112	260.2
tumor	ConvNeXt	All	60%	1152	10819	12	0.0962	0.9897	0.1754	240.42
tumor	ConvNeXt	All	70%	1782	10170	35	0.1491	0.9807	0.2588	226
tumor	ConvNeXt	All	80%	3316	9381	131	0.2612	0.962	0.4108	208.47
tumor	ConvNeXt	All	90%	1430	2439	1027	0.3696	0.582	0.4521	54.2
nerve	Virchow2	Colon	50%	136	366	7	0.2709	0.951	0.4217	24.4
nerve	Virchow2	Colon	60%	131	250	13	0.3438	0.9097	0.499	16.67
nerve	Virchow2	Colon	70%	129	168	16	0.4343	0.8897	0.5837	11.2
nerve	Virchow2	Colon	75%	127	133	20	0.4885	0.8639	0.6241	8.87
nerve	Virchow2	Colon	80%	123	103	25	0.5442	0.8311	0.6578	6.87
nerve	Virchow2	Colon	85%	113	69	40	0.6209	0.7386	0.6746	4.6
nerve	Virchow2	Colon	90%	89	29	75	0.7542	0.5427	0.6312	1.93
nerve	Virchow2	Pancreas	50%	157	172	26	0.4772	0.8579	0.6133	11.47
nerve	Virchow2	Pancreas	60%	154	125	30	0.552	0.837	0.6652	8.33
nerve	Virchow2	Pancreas	70%	148	63	38	0.7014	0.7957	0.7456	4.2
nerve	Virchow2	Pancreas	75%	140	46	47	0.7527	0.7487	0.7507	3.07
nerve	Virchow2	Pancreas	80%	130	34	60	0.7927	0.6842	0.7345	2.27
nerve	Virchow2	Pancreas	85%	124	21	79	0.8552	0.6108	0.7126	1.4
nerve	Virchow2	Pancreas	90%	102	9	109	0.9189	0.4834	0.6335	0.6
nerve	Virchow2	Prostate	50%	137	73	3	0.6524	0.9786	0.7829	4.87
nerve	Virchow2	Prostate	60%	136	50	6	0.7312	0.9577	0.8293	3.33

nerve	Virchow2	Prostate	70%	135	34	8	0.7988	0.9441	0.8654	2.27
nerve	Virchow2	Prostate	75%	137	28	9	0.8303	0.9384	0.881	1.87
nerve	Virchow2	Prostate	80%	138	13	17	0.9139	0.8903	0.902	0.87
nerve	Virchow2	Prostate	85%	141	7	33	0.9527	0.8103	0.8758	0.47
nerve	Virchow2	Prostate	90%	93	3	92	0.9688	0.5027	0.6619	0.2
nerve	Virchow2	All	50%	430	611	36	0.4131	0.9227	0.5707	13.58
nerve	Virchow2	All	60%	421	425	49	0.4976	0.8957	0.6398	9.44
nerve	Virchow2	All	70%	412	265	62	0.6086	0.8692	0.7159	5.89
nerve	Virchow2	All	75%	404	207	76	0.6612	0.8417	0.7406	4.6
nerve	Virchow2	All	80%	391	150	102	0.7227	0.7931	0.7563	3.33
nerve	Virchow2	All	85%	378	97	152	0.7958	0.7132	0.7522	2.16
nerve	Virchow2	All	90%	284	41	276	0.8738	0.5071	0.6418	0.91
nerve	UNI	Colon	50%	137	314	6	0.3038	0.958	0.4613	20.93
nerve	UNI	Colon	60%	135	167	13	0.447	0.9122	0.6	11.13
nerve	UNI	Colon	70%	129	64	30	0.6684	0.8113	0.733	4.27
nerve	UNI	Colon	75%	118	30	42	0.7973	0.7375	0.7662	2
nerve	UNI	Colon	80%	101	12	63	0.8938	0.6159	0.7292	0.8
nerve	UNI	Colon	85%	83	6	82	0.9326	0.503	0.6535	0.4
nerve	UNI	Colon	90%	68	3	105	0.9577	0.3931	0.5574	0.2
nerve	UNI	Pancreas	50%	160	141	34	0.5316	0.8247	0.6465	9.4
nerve	UNI	Pancreas	60%	153	71	46	0.683	0.7688	0.7234	4.73
nerve	UNI	Pancreas	70%	135	24	63	0.8491	0.6818	0.7563	1.6
nerve	UNI	Pancreas	75%	123	13	77	0.9044	0.615	0.7321	0.87
nerve	UNI	Pancreas	80%	106	10	93	0.9138	0.5327	0.673	0.67
nerve	UNI	Pancreas	85%	84	5	111	0.9438	0.4308	0.5915	0.33
nerve	UNI	Pancreas	90%	62	1	141	0.9841	0.3054	0.4662	0.07
nerve	UNI	Prostate	50%	139	38	8	0.7853	0.9456	0.858	2.53
nerve	UNI	Prostate	60%	141	13	9	0.9156	0.94	0.9276	0.87
nerve	UNI	Prostate	70%	143	4	11	0.9728	0.9286	0.9502	0.27
nerve	UNI	Prostate	75%	139	1	15	0.9929	0.9026	0.9456	0.07
nerve	UNI	Prostate	80%	147	1	18	0.9932	0.8909	0.9393	0.07
nerve	UNI	Prostate	85%	151	1	26	0.9934	0.8531	0.9179	0.07
nerve	UNI	Prostate	90%	115	0	67	1	0.6319	0.7744	0
nerve	UNI	All	50%	436	493	48	0.4693	0.9008	0.6171	10.96
nerve	UNI	All	60%	429	251	68	0.6309	0.8632	0.729	5.58
nerve	UNI	All	70%	407	92	104	0.8156	0.7965	0.8059	2.04
nerve	UNI	All	75%	380	44	134	0.8962	0.7393	0.8102	0.98
nerve	UNI	All	80%	354	23	174	0.939	0.6705	0.7823	0.51
nerve	UNI	All	85%	318	12	219	0.9636	0.5922	0.7336	0.27
nerve	UNI	All	90%	245	4	313	0.9839	0.4391	0.6072	0.09
nerve	EfficientNet	All	50%	337	5721	81	0.0556	0.8062	0.1041	127.13
nerve	EfficientNet	All	60%	341	3386	108	0.0915	0.7595	0.1633	75.24

nerve	EfficientNet	All	70%	314	2004	171	0.1355	0.6474	0.224	44.53
nerve	EfficientNet	All	80%	257	1031	268	0.1995	0.4895	0.2835	22.91
nerve	EfficientNet	All	90%	56	98	480	0.3636	0.1045	0.1623	2.18
nerve	ConvNeXt	All	50%	346	8223	51	0.0404	0.8715	0.0772	182.73
nerve	ConvNeXt	All	60%	351	5673	70	0.0583	0.8337	0.1089	126.07
nerve	ConvNeXt	All	70%	347	3643	94	0.087	0.7868	0.1566	80.96
nerve	ConvNeXt	All	80%	312	1817	169	0.1465	0.6486	0.2391	40.38
nerve	ConvNeXt	All	90%	0	0	529	0	0	0	0

Table S6a. Per-class summary results for the Baseline approach

Class	Model	Organ	Threshold	TP	FP	FN	Precision	Recall	F1	Avg_FP_per_Slide
PNI	Virchow2	Colon	50%	130	171	9	0.4319	0.9353	0.5909	11.4
PNI	Virchow2	Colon	60%	125	87	15	0.5896	0.8929	0.7102	5.8
PNI	Virchow2	Colon	70%	111	41	29	0.7303	0.7929	0.7603	2.73
PNI	Virchow2	Colon	75%	90	21	57	0.8108	0.6122	0.6977	1.4
PNI	Virchow2	Colon	80%	52	11	96	0.8254	0.3514	0.4929	0.73
PNI	Virchow2	Colon	85%	15	2	133	0.8824	0.1014	0.1818	0.13
PNI	Virchow2	Colon	90%	0	0	151	0	0	0	0
PNI	Virchow2	Pancreas	50%	215	425	14	0.3359	0.9389	0.4948	28.33
PNI	Virchow2	Pancreas	60%	202	283	28	0.4165	0.8783	0.565	18.87
PNI	Virchow2	Pancreas	70%	168	156	71	0.5185	0.7029	0.5968	10.4
PNI	Virchow2	Pancreas	75%	149	102	97	0.5936	0.6057	0.5996	6.8
PNI	Virchow2	Pancreas	80%	92	55	156	0.6259	0.371	0.4658	3.67
PNI	Virchow2	Pancreas	85%	43	15	202	0.7414	0.1755	0.2838	1
PNI	Virchow2	Pancreas	90%	8	1	235	0.8889	0.0329	0.0635	0.07
PNI	Virchow2	Prostate	50%	271	122	7	0.6896	0.9748	0.8077	8.13
PNI	Virchow2	Prostate	60%	266	87	11	0.7535	0.9603	0.8444	5.8
PNI	Virchow2	Prostate	70%	256	57	22	0.8179	0.9209	0.8663	3.8
PNI	Virchow2	Prostate	75%	235	34	42	0.8736	0.8484	0.8608	2.27
PNI	Virchow2	Prostate	80%	179	14	102	0.9275	0.637	0.7553	0.93
PNI	Virchow2	Prostate	85%	73	1	215	0.9865	0.2535	0.4033	0.07
PNI	Virchow2	Prostate	90%	4	0	286	1	0.0138	0.0272	0
PNI	Virchow2	All	50%	616	718	30	0.4618	0.9536	0.6222	15.96
PNI	Virchow2	All	60%	593	457	54	0.5648	0.9165	0.6989	10.16
PNI	Virchow2	All	70%	535	254	122	0.6781	0.8143	0.74	5.64
PNI	Virchow2	All	75%	474	157	196	0.7512	0.7075	0.7287	3.49
PNI	Virchow2	All	80%	323	80	354	0.8015	0.4771	0.5981	1.78
PNI	Virchow2	All	85%	131	18	550	0.8792	0.1924	0.3157	0.4
PNI	Virchow2	All	90%	12	1	672	0.9231	0.0175	0.0344	0.02
PNI	UNI	Colon	50%	128	165	11	0.4369	0.9209	0.5926	11
PNI	UNI	Colon	60%	126	82	15	0.6058	0.8936	0.7221	5.47
PNI	UNI	Colon	70%	116	15	29	0.8855	0.8	0.8406	1

PNI	UNI	Colon	75%	88	4	59	0.9565	0.5986	0.7364	0.27
PNI	UNI	Colon	80%	41	2	108	0.9535	0.2752	0.4271	0.13
PNI	UNI	Colon	85%	0	0	151	0	0	0	0
PNI	UNI	Colon	90%	0	0	151	0	0	0	0
PNI	UNI	Pancreas	50%	215	400	17	0.3496	0.9267	0.5077	26.67
PNI	UNI	Pancreas	60%	205	195	27	0.5125	0.8836	0.6487	13
PNI	UNI	Pancreas	70%	181	44	59	0.8044	0.7542	0.7785	2.93
PNI	UNI	Pancreas	75%	167	11	79	0.9382	0.6789	0.7877	0.73
PNI	UNI	Pancreas	80%	82	0	165	1	0.332	0.4985	0
PNI	UNI	Pancreas	85%	3	0	238	1	0.0124	0.0246	0
PNI	UNI	Pancreas	90%	0	0	242	0	0	0	0
PNI	UNI	Prostate	50%	261	97	18	0.7291	0.9355	0.8195	6.47
PNI	UNI	Prostate	60%	258	60	20	0.8113	0.9281	0.8658	4
PNI	UNI	Prostate	70%	240	41	39	0.8541	0.8602	0.8571	2.73
PNI	UNI	Prostate	75%	204	33	75	0.8608	0.7312	0.7907	2.2
PNI	UNI	Prostate	80%	118	16	167	0.8806	0.414	0.5632	1.07
PNI	UNI	Prostate	85%	17	1	271	0.9444	0.059	0.1111	0.07
PNI	UNI	Prostate	90%	0	0	291	0	0	0	0
PNI	UNI	All	50%	604	662	46	0.4771	0.9292	0.6305	14.71
PNI	UNI	All	60%	589	337	62	0.6361	0.9048	0.747	7.49
PNI	UNI	All	70%	537	100	127	0.843	0.8087	0.8255	2.22
PNI	UNI	All	75%	459	48	213	0.9053	0.683	0.7786	1.07
PNI	UNI	All	80%	241	18	440	0.9305	0.3539	0.5128	0.4
PNI	UNI	All	85%	20	1	660	0.9524	0.0294	0.0571	0.02
PNI	UNI	All	90%	0	0	684	0	0	0	0
PNI	EfficientNet	Colon	50%	80	6328	8	0.0125	0.9091	0.0246	421.87
PNI	EfficientNet	Colon	60%	92	4502	17	0.02	0.844	0.0391	300.13
PNI	EfficientNet	Colon	70%	108	2718	24	0.0382	0.8182	0.073	181.2
PNI	EfficientNet	Colon	80%	102	1256	50	0.0751	0.6711	0.1351	83.73
PNI	EfficientNet	Colon	90%	72	292	114	0.1978	0.3871	0.2618	19.47
PNI	EfficientNet	Pancreas	50%	93	9556	5	0.0096	0.949	0.0191	637.07
PNI	EfficientNet	Pancreas	60%	122	7294	12	0.0165	0.9104	0.0323	486.27
PNI	EfficientNet	Pancreas	70%	149	4709	20	0.0307	0.8817	0.0593	313.93
PNI	EfficientNet	Pancreas	80%	151	2105	49	0.0669	0.755	0.123	140.33
PNI	EfficientNet	Pancreas	90%	136	374	108	0.2667	0.5574	0.3607	24.93
PNI	EfficientNet	Prostate	50%	204	2781	44	0.0683	0.8226	0.1262	185.4
PNI	EfficientNet	Prostate	60%	203	1514	54	0.1182	0.7899	0.2057	100.93
PNI	EfficientNet	Prostate	70%	196	788	71	0.1992	0.7341	0.3133	52.53
PNI	EfficientNet	Prostate	80%	176	400	102	0.3056	0.6331	0.4122	26.67
PNI	EfficientNet	Prostate	90%	126	127	171	0.498	0.4242	0.4582	8.47
PNI	EfficientNet	All	50%	377	18665	57	0.0198	0.8687	0.0387	414.78
PNI	EfficientNet	All	60%	417	13310	83	0.0304	0.834	0.0586	295.78

PNI	EfficientNet	All	70%	453	8215	115	0.0523	0.7975	0.0981	182.56
PNI	EfficientNet	All	80%	429	3761	201	0.1024	0.681	0.178	83.58
PNI	EfficientNet	All	90%	334	793	393	0.2964	0.4594	0.3603	17.62
PNI	ConvNeXt	Colon	50%	77	4240	15	0.0178	0.837	0.0349	282.67
PNI	ConvNeXt	Colon	60%	84	3130	18	0.0261	0.8235	0.0507	208.67
PNI	ConvNeXt	Colon	70%	99	2044	26	0.0462	0.792	0.0873	136.27
PNI	ConvNeXt	Colon	80%	103	1037	51	0.0904	0.6688	0.1592	69.13
PNI	ConvNeXt	Colon	90%	64	155	134	0.2922	0.3232	0.307	10.33
PNI	ConvNeXt	Pancreas	50%	114	8752	4	0.0129	0.9661	0.0254	583.47
PNI	ConvNeXt	Pancreas	60%	137	6978	8	0.0193	0.9448	0.0377	465.2
PNI	ConvNeXt	Pancreas	70%	148	4852	15	0.0296	0.908	0.0573	323.47
PNI	ConvNeXt	Pancreas	80%	157	2277	35	0.0645	0.8177	0.1196	151.8
PNI	ConvNeXt	Pancreas	90%	116	195	137	0.373	0.4585	0.4113	13
PNI	ConvNeXt	Prostate	50%	213	985	52	0.1778	0.8038	0.2912	65.67
PNI	ConvNeXt	Prostate	60%	203	649	67	0.2383	0.7519	0.3619	43.27
PNI	ConvNeXt	Prostate	70%	195	393	85	0.3316	0.6964	0.4493	26.2
PNI	ConvNeXt	Prostate	80%	157	211	131	0.4266	0.5451	0.4787	14.07
PNI	ConvNeXt	Prostate	90%	52	26	257	0.6667	0.1683	0.2687	1.73
PNI	ConvNeXt	All	50%	404	13977	71	0.0281	0.8505	0.0544	310.6
PNI	ConvNeXt	All	60%	424	10757	93	0.0379	0.8201	0.0725	239.04
PNI	ConvNeXt	All	70%	442	7289	126	0.0572	0.7782	0.1065	161.98
PNI	ConvNeXt	All	80%	417	3525	217	0.1058	0.6577	0.1823	78.33
PNI	ConvNeXt	All	90%	232	376	528	0.3816	0.3053	0.3392	8.36
tumor	Virchow2	Colon	50%	451	85	1	0.8414	0.9978	0.913	5.67
tumor	Virchow2	Colon	60%	484	60	4	0.8897	0.9918	0.938	4
tumor	Virchow2	Colon	70%	614	41	7	0.9374	0.9887	0.9624	2.73
tumor	Virchow2	Colon	75%	662	35	10	0.9498	0.9851	0.9671	2.33
tumor	Virchow2	Colon	80%	766	27	13	0.966	0.9833	0.9746	1.8
tumor	Virchow2	Colon	85%	913	20	20	0.9786	0.9786	0.9786	1.33
tumor	Virchow2	Colon	90%	997	7	43	0.993	0.9587	0.9755	0.47
tumor	Virchow2	Pancreas	50%	600	190	10	0.7595	0.9836	0.8571	12.67
tumor	Virchow2	Pancreas	60%	669	134	12	0.8331	0.9824	0.9016	8.93
tumor	Virchow2	Pancreas	70%	734	90	21	0.8908	0.9722	0.9297	6
tumor	Virchow2	Pancreas	75%	794	72	27	0.9169	0.9671	0.9413	4.8
tumor	Virchow2	Pancreas	80%	810	44	55	0.9485	0.9364	0.9424	2.93
tumor	Virchow2	Pancreas	85%	861	25	88	0.9718	0.9073	0.9384	1.67
tumor	Virchow2	Pancreas	90%	875	12	138	0.9865	0.8638	0.9211	0.8
tumor	Virchow2	Prostate	50%	632	132	25	0.8272	0.9619	0.8895	8.8
tumor	Virchow2	Prostate	60%	665	90	29	0.8808	0.9582	0.9179	6
tumor	Virchow2	Prostate	70%	706	53	40	0.9302	0.9464	0.9382	3.53
tumor	Virchow2	Prostate	75%	720	40	49	0.9474	0.9363	0.9418	2.67
tumor	Virchow2	Prostate	80%	789	33	60	0.9599	0.9293	0.9443	2.2

tumor	Virchow2	Prostate	85%	901	21	75	0.9772	0.9232	0.9494	1.4
tumor	Virchow2	Prostate	90%	1080	12	113	0.989	0.9053	0.9453	0.8
tumor	Virchow2	All	50%	1683	407	36	0.8053	0.9791	0.8837	9.04
tumor	Virchow2	All	60%	1818	284	45	0.8649	0.9758	0.917	6.31
tumor	Virchow2	All	70%	2054	184	68	0.9178	0.968	0.9422	4.09
tumor	Virchow2	All	75%	2176	147	86	0.9367	0.962	0.9492	3.27
tumor	Virchow2	All	80%	2365	104	128	0.9579	0.9487	0.9532	2.31
tumor	Virchow2	All	85%	2675	66	183	0.9759	0.936	0.9555	1.47
tumor	Virchow2	All	90%	2952	31	294	0.9896	0.9094	0.9478	0.69
tumor	UNI	Colon	50%	488	125	2	0.7961	0.9959	0.8849	8.33
tumor	UNI	Colon	60%	554	85	4	0.867	0.9928	0.9256	5.67
tumor	UNI	Colon	70%	702	49	5	0.9348	0.9929	0.963	3.27
tumor	UNI	Colon	75%	788	41	8	0.9505	0.9899	0.9698	2.73
tumor	UNI	Colon	80%	908	26	10	0.9722	0.9891	0.9806	1.73
tumor	UNI	Colon	85%	1042	15	23	0.9858	0.9784	0.9821	1
tumor	UNI	Colon	90%	1079	8	58	0.9926	0.949	0.9703	0.53
tumor	UNI	Pancreas	50%	643	209	9	0.7547	0.9862	0.8551	13.93
tumor	UNI	Pancreas	60%	732	191	13	0.7931	0.9826	0.8777	12.73
tumor	UNI	Pancreas	70%	861	146	21	0.855	0.9762	0.9116	9.73
tumor	UNI	Pancreas	75%	927	118	34	0.8871	0.9646	0.9242	7.87
tumor	UNI	Pancreas	80%	949	83	47	0.9196	0.9528	0.9359	5.53
tumor	UNI	Pancreas	85%	952	46	80	0.9539	0.9225	0.9379	3.07
tumor	UNI	Pancreas	90%	905	15	141	0.9837	0.8652	0.9207	1
tumor	UNI	Prostate	50%	627	232	20	0.7299	0.9691	0.8327	15.47
tumor	UNI	Prostate	60%	649	139	24	0.8236	0.9643	0.8884	9.27
tumor	UNI	Prostate	70%	714	78	29	0.9015	0.961	0.9303	5.2
tumor	UNI	Prostate	75%	771	60	38	0.9278	0.953	0.9402	4
tumor	UNI	Prostate	80%	844	41	45	0.9537	0.9494	0.9515	2.73
tumor	UNI	Prostate	85%	1006	25	58	0.9758	0.9455	0.9604	1.67
tumor	UNI	Prostate	90%	1267	14	89	0.9891	0.9344	0.9609	0.93
tumor	UNI	All	50%	1758	566	31	0.7565	0.9827	0.8549	12.58
tumor	UNI	All	60%	1935	415	41	0.8234	0.9793	0.8946	9.22
tumor	UNI	All	70%	2277	273	55	0.8929	0.9764	0.9328	6.07
tumor	UNI	All	75%	2486	219	80	0.919	0.9688	0.9433	4.87
tumor	UNI	All	80%	2701	150	102	0.9474	0.9636	0.9554	3.33
tumor	UNI	All	85%	3000	86	161	0.9721	0.9491	0.9605	1.91
tumor	UNI	All	90%	3251	37	288	0.9887	0.9186	0.9524	0.82
tumor	EfficientNet	All	50%	917	17527	4	0.0497	0.9957	0.0947	389.49
tumor	EfficientNet	All	60%	1634	15089	15	0.0977	0.9909	0.1779	335.31
tumor	EfficientNet	All	70%	2650	12441	48	0.1756	0.9822	0.2979	276.47
tumor	EfficientNet	All	80%	3789	9997	154	0.2748	0.9609	0.4274	222.16
tumor	EfficientNet	All	90%	3332	5814	535	0.3643	0.8616	0.5121	129.2

tumor	ConvNeXt	All	50%	693	14423	3	0.0458	0.9957	0.0877	320.51
tumor	ConvNeXt	All	60%	983	12375	10	0.0736	0.9899	0.137	275
tumor	ConvNeXt	All	70%	1867	11021	23	0.1449	0.9878	0.2527	244.91
tumor	ConvNeXt	All	80%	3097	9142	101	0.253	0.9684	0.4012	203.16
tumor	ConvNeXt	All	90%	2598	4029	714	0.392	0.7844	0.5228	89.53
nerve	Virchow2	Colon	50%	138	203	5	0.4047	0.965	0.5702	13.53
nerve	Virchow2	Colon	60%	137	120	6	0.5331	0.958	0.685	8
nerve	Virchow2	Colon	70%	134	81	10	0.6233	0.9306	0.7465	5.4
nerve	Virchow2	Colon	75%	128	56	18	0.6957	0.8767	0.7758	3.73
nerve	Virchow2	Colon	80%	125	33	24	0.7911	0.8389	0.8143	2.2
nerve	Virchow2	Colon	85%	119	23	40	0.838	0.7484	0.7907	1.53
nerve	Virchow2	Colon	90%	99	8	71	0.9252	0.5824	0.7148	0.53
nerve	Virchow2	Pancreas	50%	159	134	25	0.5427	0.8641	0.6667	8.93
nerve	Virchow2	Pancreas	60%	155	84	30	0.6485	0.8378	0.7311	5.6
nerve	Virchow2	Pancreas	70%	144	51	40	0.7385	0.7826	0.7599	3.4
nerve	Virchow2	Pancreas	75%	141	33	49	0.8103	0.7421	0.7747	2.2
nerve	Virchow2	Pancreas	80%	130	24	64	0.8442	0.6701	0.7471	1.6
nerve	Virchow2	Pancreas	85%	120	15	78	0.8889	0.6061	0.7207	1
nerve	Virchow2	Pancreas	90%	92	6	111	0.9388	0.4532	0.6113	0.4
nerve	Virchow2	Prostate	50%	136	57	5	0.7047	0.9645	0.8144	3.8
nerve	Virchow2	Prostate	60%	136	35	5	0.7953	0.9645	0.8718	2.33
nerve	Virchow2	Prostate	70%	139	20	5	0.8742	0.9653	0.9175	1.33
nerve	Virchow2	Prostate	75%	140	14	5	0.9091	0.9655	0.9365	0.93
nerve	Virchow2	Prostate	80%	140	8	7	0.9459	0.9524	0.9492	0.53
nerve	Virchow2	Prostate	85%	144	5	15	0.9664	0.9057	0.9351	0.33
nerve	Virchow2	Prostate	90%	143	3	35	0.9795	0.8034	0.8827	0.2
nerve	Virchow2	All	50%	433	394	35	0.5236	0.9252	0.6687	8.76
nerve	Virchow2	All	60%	428	239	41	0.6417	0.9126	0.7535	5.31
nerve	Virchow2	All	70%	417	152	55	0.7329	0.8835	0.8012	3.38
nerve	Virchow2	All	75%	409	103	72	0.7988	0.8503	0.8238	2.29
nerve	Virchow2	All	80%	395	65	95	0.8587	0.8061	0.8316	1.44
nerve	Virchow2	All	85%	383	43	133	0.8991	0.7422	0.8132	0.96
nerve	Virchow2	All	90%	334	17	217	0.9516	0.6062	0.7406	0.38
nerve	UNI	Colon	50%	141	277	3	0.3373	0.9792	0.5018	18.47
nerve	UNI	Colon	60%	142	154	3	0.4797	0.9793	0.644	10.27
nerve	UNI	Colon	70%	142	83	4	0.6311	0.9726	0.7655	5.53
nerve	UNI	Colon	75%	137	48	10	0.7405	0.932	0.8253	3.2
nerve	UNI	Colon	80%	135	33	14	0.8036	0.906	0.8517	2.2
nerve	UNI	Colon	85%	130	21	23	0.8609	0.8497	0.8553	1.4
nerve	UNI	Colon	90%	129	15	42	0.8958	0.7544	0.819	1
nerve	UNI	Pancreas	50%	159	247	24	0.3916	0.8689	0.5399	16.47
nerve	UNI	Pancreas	60%	155	177	30	0.4669	0.8378	0.5996	11.8

nerve	UNI	Pancreas	70%	154	101	35	0.6039	0.8148	0.6937	6.73
nerve	UNI	Pancreas	75%	151	72	43	0.6771	0.7784	0.7242	4.8
nerve	UNI	Pancreas	80%	144	53	52	0.731	0.7347	0.7328	3.53
nerve	UNI	Pancreas	85%	132	29	65	0.8199	0.6701	0.7374	1.93
nerve	UNI	Pancreas	90%	104	12	94	0.8966	0.5253	0.6624	0.8
nerve	UNI	Prostate	50%	136	28	5	0.8293	0.9645	0.8918	1.87
nerve	UNI	Prostate	60%	137	20	6	0.8726	0.958	0.9133	1.33
nerve	UNI	Prostate	70%	143	8	8	0.947	0.947	0.947	0.53
nerve	UNI	Prostate	75%	144	3	9	0.9796	0.9412	0.96	0.2
nerve	UNI	Prostate	80%	139	2	13	0.9858	0.9145	0.9488	0.13
nerve	UNI	Prostate	85%	140	2	18	0.9859	0.8861	0.9333	0.13
nerve	UNI	Prostate	90%	145	1	29	0.9932	0.8333	0.9063	0.07
nerve	UNI	All	50%	436	552	32	0.4413	0.9316	0.5989	12.27
nerve	UNI	All	60%	434	351	39	0.5529	0.9175	0.69	7.8
nerve	UNI	All	70%	439	192	47	0.6957	0.9033	0.786	4.27
nerve	UNI	All	75%	432	123	62	0.7784	0.8745	0.8236	2.73
nerve	UNI	All	80%	418	88	79	0.8261	0.841	0.8335	1.96
nerve	UNI	All	85%	402	52	106	0.8855	0.7913	0.8358	1.16
nerve	UNI	All	90%	378	28	165	0.931	0.6961	0.7966	0.62
nerve	EfficientNet	All	50%	298	22230	36	0.0132	0.8922	0.0261	494
nerve	EfficientNet	All	60%	324	16587	53	0.0192	0.8594	0.0375	368.6
nerve	EfficientNet	All	70%	345	11152	66	0.03	0.8394	0.0579	247.82
nerve	EfficientNet	All	80%	362	6330	100	0.0541	0.7835	0.1012	140.67
nerve	EfficientNet	All	90%	326	2683	177	0.1083	0.6481	0.1856	59.62
nerve	ConvNeXt	All	50%	298	18210	35	0.0161	0.8949	0.0316	404.67
nerve	ConvNeXt	All	60%	325	13926	46	0.0228	0.876	0.0445	309.47
nerve	ConvNeXt	All	70%	339	9667	61	0.0339	0.8475	0.0652	214.82
nerve	ConvNeXt	All	80%	350	5708	87	0.0578	0.8009	0.1078	126.84
nerve	ConvNeXt	All	90%	309	2233	161	0.1216	0.6574	0.2052	49.62

Table S6b. Per-class summary results for the Ablation approach

Class	Model	Organ	Threshold	TP	FP	FN	Precision	Recall	F1	Avg_FP_per_Slide
PNI	Virchow2	Colon	50%	128	137	12	0.483	0.9143	0.6321	9.13
PNI	Virchow2	Colon	60%	111	40	34	0.7351	0.7655	0.75	2.67
PNI	Virchow2	Colon	70%	74	8	75	0.9024	0.4966	0.6407	0.53
PNI	Virchow2	Colon	75%	45	3	103	0.9375	0.3041	0.4592	0.2
PNI	Virchow2	Colon	80%	12	2	139	0.8571	0.0795	0.1455	0.13
PNI	Virchow2	Colon	85%	0	0	151	0	0	0	0
PNI	Virchow2	Colon	90%	0	0	151	0	0	0	0
PNI	Virchow2	Pancreas	50%	208	188	25	0.5253	0.8927	0.6614	12.53
PNI	Virchow2	Pancreas	60%	188	59	51	0.7611	0.7866	0.7737	3.93

PNI	Virchow2	Pancreas	70%	117	5	129	0.959	0.4756	0.6359	0.33
PNI	Virchow2	Pancreas	75%	82	0	164	1	0.3333	0.5	0
PNI	Virchow2	Pancreas	80%	30	0	208	1	0.1261	0.2239	0
PNI	Virchow2	Pancreas	85%	5	0	237	1	0.0207	0.0405	0
PNI	Virchow2	Pancreas	90%	0	0	242	0	0	0	0
PNI	Virchow2	Prostate	50%	268	109	11	0.7109	0.9606	0.8171	7.27
PNI	Virchow2	Prostate	60%	260	72	17	0.7831	0.9386	0.8539	4.8
PNI	Virchow2	Prostate	70%	229	37	48	0.8609	0.8267	0.8435	2.47
PNI	Virchow2	Prostate	75%	199	27	82	0.8805	0.7082	0.785	1.8
PNI	Virchow2	Prostate	80%	145	13	145	0.9177	0.5	0.6473	0.87
PNI	Virchow2	Prostate	85%	26	3	263	0.8966	0.09	0.1635	0.2
PNI	Virchow2	Prostate	90%	0	0	291	0	0	0	0
PNI	Virchow2	All	50%	604	434	48	0.5819	0.9264	0.7148	9.64
PNI	Virchow2	All	60%	559	171	102	0.7658	0.8457	0.8037	3.8
PNI	Virchow2	All	70%	420	50	252	0.8936	0.625	0.7356	1.11
PNI	Virchow2	All	75%	326	30	349	0.9157	0.483	0.6324	0.67
PNI	Virchow2	All	80%	187	15	492	0.9257	0.2754	0.4245	0.33
PNI	Virchow2	All	85%	31	3	651	0.9118	0.0455	0.0866	0.07
PNI	Virchow2	All	90%	0	0	684	0	0	0	0
PNI	UNI	Colon	50%	128	265	10	0.3257	0.9275	0.4821	17.67
PNI	UNI	Colon	60%	126	99	14	0.56	0.9	0.6904	6.6
PNI	UNI	Colon	70%	107	22	35	0.8295	0.7535	0.7897	1.47
PNI	UNI	Colon	75%	82	7	63	0.9213	0.5655	0.7009	0.47
PNI	UNI	Colon	80%	28	0	124	1	0.1842	0.3111	0
PNI	UNI	Colon	85%	1	0	150	1	0.0066	0.0132	0
PNI	UNI	Colon	90%	0	0	151	0	0	0	0
PNI	UNI	Pancreas	50%	213	275	16	0.4365	0.9301	0.5941	18.33
PNI	UNI	Pancreas	60%	204	119	27	0.6316	0.8831	0.7365	7.93
PNI	UNI	Pancreas	70%	179	24	60	0.8818	0.749	0.81	1.6
PNI	UNI	Pancreas	75%	135	2	109	0.9854	0.5533	0.7087	0.13
PNI	UNI	Pancreas	80%	69	0	172	1	0.2863	0.4452	0
PNI	UNI	Pancreas	85%	3	0	238	1	0.0124	0.0246	0
PNI	UNI	Pancreas	90%	0	0	242	0	0	0	0
PNI	UNI	Prostate	50%	265	119	12	0.6901	0.9567	0.8018	7.93
PNI	UNI	Prostate	60%	258	72	19	0.7818	0.9314	0.8501	4.8
PNI	UNI	Prostate	70%	231	43	49	0.8431	0.825	0.8339	2.87
PNI	UNI	Prostate	75%	188	33	91	0.8507	0.6738	0.752	2.2
PNI	UNI	Prostate	80%	85	17	200	0.8333	0.2982	0.4393	1.13
PNI	UNI	Prostate	85%	5	0	285	1	0.0172	0.0339	0
PNI	UNI	Prostate	90%	0	0	291	0	0	0	0
PNI	UNI	All	50%	606	659	38	0.4791	0.941	0.6349	14.64
PNI	UNI	All	60%	588	290	60	0.6697	0.9074	0.7706	6.44

PNI	UNI	All	70%	517	89	144	0.8531	0.7821	0.8161	1.98
PNI	UNI	All	75%	405	42	263	0.906	0.6063	0.7265	0.93
PNI	UNI	All	80%	182	17	496	0.9146	0.2684	0.4151	0.38
PNI	UNI	All	85%	9	0	673	1	0.0132	0.026	0
PNI	UNI	All	90%	0	0	684	0	0	0	0
tumor	Virchow2	Colon	50%	700	44	26	0.9409	0.9642	0.9524	2.93
tumor	Virchow2	Colon	60%	851	26	44	0.9704	0.9508	0.9605	1.73
tumor	Virchow2	Colon	70%	896	15	86	0.9835	0.9124	0.9466	1
tumor	Virchow2	Colon	75%	878	8	115	0.991	0.8842	0.9345	0.53
tumor	Virchow2	Colon	80%	796	7	156	0.9913	0.8361	0.9071	0.47
tumor	Virchow2	Colon	85%	629	3	235	0.9953	0.728	0.8409	0.2
tumor	Virchow2	Colon	90%	377	1	299	0.9974	0.5577	0.7154	0.07
tumor	Virchow2	Pancreas	50%	679	97	20	0.875	0.9714	0.9207	6.47
tumor	Virchow2	Pancreas	60%	775	55	50	0.9337	0.9394	0.9366	3.67
tumor	Virchow2	Pancreas	70%	803	13	118	0.9841	0.8719	0.9246	0.87
tumor	Virchow2	Pancreas	75%	742	8	178	0.9893	0.8065	0.8886	0.53
tumor	Virchow2	Pancreas	80%	656	5	246	0.9924	0.7273	0.8394	0.33
tumor	Virchow2	Pancreas	85%	540	2	309	0.9963	0.636	0.7764	0.13
tumor	Virchow2	Pancreas	90%	259	0	386	1	0.4016	0.573	0
tumor	Virchow2	Prostate	50%	665	45	30	0.9366	0.9568	0.9466	3
tumor	Virchow2	Prostate	60%	791	38	46	0.9542	0.945	0.9496	2.53
tumor	Virchow2	Prostate	70%	1031	14	87	0.9866	0.9222	0.9533	0.93
tumor	Virchow2	Prostate	75%	1071	6	130	0.9944	0.8918	0.9403	0.4
tumor	Virchow2	Prostate	80%	1091	5	194	0.9954	0.849	0.9164	0.33
tumor	Virchow2	Prostate	85%	1095	1	276	0.9991	0.7987	0.8877	0.07
tumor	Virchow2	Prostate	90%	600	0	410	1	0.5941	0.7453	0
tumor	Virchow2	All	50%	2044	186	76	0.9166	0.9642	0.9398	4.13
tumor	Virchow2	All	60%	2417	119	140	0.9531	0.9452	0.9491	2.64
tumor	Virchow2	All	70%	2730	42	291	0.9848	0.9037	0.9425	0.93
tumor	Virchow2	All	75%	2691	22	423	0.9919	0.8642	0.9236	0.49
tumor	Virchow2	All	80%	2543	17	596	0.9934	0.8101	0.8924	0.38
tumor	Virchow2	All	85%	2264	6	820	0.9974	0.7341	0.8457	0.13
tumor	Virchow2	All	90%	1236	1	1095	0.9992	0.5302	0.6928	0.02
tumor	UNI	Colon	50%	607	106	8	0.8513	0.987	0.9142	7.07
tumor	UNI	Colon	60%	733	69	15	0.914	0.9799	0.9458	4.6
tumor	UNI	Colon	70%	948	43	25	0.9566	0.9743	0.9654	2.87
tumor	UNI	Colon	75%	1054	31	31	0.9714	0.9714	0.9714	2.07
tumor	UNI	Colon	80%	1150	27	45	0.9771	0.9623	0.9696	1.8
tumor	UNI	Colon	85%	1153	13	63	0.9889	0.9482	0.9681	0.87
tumor	UNI	Colon	90%	1085	4	97	0.9963	0.9179	0.9555	0.27
tumor	UNI	Pancreas	50%	618	238	4	0.722	0.9936	0.8363	15.87
tumor	UNI	Pancreas	60%	676	204	9	0.7682	0.9869	0.8639	13.6

tumor	UNI	Pancreas	70%	762	143	15	0.842	0.9807	0.9061	9.53
tumor	UNI	Pancreas	75%	833	103	29	0.89	0.9664	0.9266	6.87
tumor	UNI	Pancreas	80%	934	83	45	0.9184	0.954	0.9359	5.53
tumor	UNI	Pancreas	85%	1033	59	84	0.946	0.9248	0.9353	3.93
tumor	UNI	Pancreas	90%	973	32	154	0.9682	0.8634	0.9128	2.13
tumor	UNI	Prostate	50%	682	168	20	0.8024	0.9715	0.8789	11.2
tumor	UNI	Prostate	60%	744	97	30	0.8847	0.9612	0.9214	6.47
tumor	UNI	Prostate	70%	843	56	38	0.9377	0.9569	0.9472	3.73
tumor	UNI	Prostate	75%	934	44	48	0.955	0.9511	0.9531	2.93
tumor	UNI	Prostate	80%	1029	28	61	0.9735	0.944	0.9585	1.87
tumor	UNI	Prostate	85%	1212	18	72	0.9854	0.9439	0.9642	1.2
tumor	UNI	Prostate	90%	1424	6	108	0.9958	0.9295	0.9615	0.4
tumor	UNI	All	50%	1907	512	32	0.7883	0.9835	0.8752	11.38
tumor	UNI	All	60%	2153	370	54	0.8533	0.9755	0.9104	8.22
tumor	UNI	All	70%	2553	242	78	0.9134	0.9704	0.941	5.38
tumor	UNI	All	75%	2821	178	108	0.9406	0.9631	0.9518	3.96
tumor	UNI	All	80%	3113	138	151	0.9576	0.9537	0.9556	3.07
tumor	UNI	All	85%	3398	90	219	0.9742	0.9395	0.9565	2
tumor	UNI	All	90%	3482	42	359	0.9881	0.9065	0.9456	0.93
nerve	Virchow2	Colon	50%	140	250	3	0.359	0.979	0.5253	16.67
nerve	Virchow2	Colon	60%	135	169	9	0.4441	0.9375	0.6027	11.27
nerve	Virchow2	Colon	70%	133	100	11	0.5708	0.9236	0.7056	6.67
nerve	Virchow2	Colon	75%	133	81	15	0.6215	0.8986	0.7348	5.4
nerve	Virchow2	Colon	80%	127	59	24	0.6828	0.8411	0.7537	3.93
nerve	Virchow2	Colon	85%	116	42	38	0.7342	0.7532	0.7436	2.8
nerve	Virchow2	Colon	90%	100	25	68	0.8	0.5952	0.6826	1.67
nerve	Virchow2	Pancreas	50%	159	121	25	0.5679	0.8641	0.6853	8.07
nerve	Virchow2	Pancreas	60%	148	68	37	0.6852	0.8	0.7382	4.53
nerve	Virchow2	Pancreas	70%	137	33	54	0.8059	0.7173	0.759	2.2
nerve	Virchow2	Pancreas	75%	133	29	63	0.821	0.6786	0.743	1.93
nerve	Virchow2	Pancreas	80%	125	20	71	0.8621	0.6378	0.7331	1.33
nerve	Virchow2	Pancreas	85%	112	13	85	0.896	0.5685	0.6957	0.87
nerve	Virchow2	Pancreas	90%	86	6	113	0.9348	0.4322	0.5911	0.4
nerve	Virchow2	Prostate	50%	138	142	1	0.4929	0.9928	0.6587	9.47
nerve	Virchow2	Prostate	60%	137	108	3	0.5592	0.9786	0.7117	7.2
nerve	Virchow2	Prostate	70%	137	53	4	0.7211	0.9716	0.8278	3.53
nerve	Virchow2	Prostate	75%	140	32	4	0.814	0.9722	0.8861	2.13
nerve	Virchow2	Prostate	80%	141	22	8	0.865	0.9463	0.9038	1.47
nerve	Virchow2	Prostate	85%	141	6	18	0.9592	0.8868	0.9216	0.4
nerve	Virchow2	Prostate	90%	136	2	43	0.9855	0.7598	0.858	0.13
nerve	Virchow2	All	50%	437	513	29	0.46	0.9378	0.6172	11.4
nerve	Virchow2	All	60%	420	345	49	0.549	0.8955	0.6807	7.67

nerve	Virchow2	All	70%	407	186	69	0.6863	0.855	0.7615	4.13
nerve	Virchow2	All	75%	406	142	82	0.7409	0.832	0.7838	3.16
nerve	Virchow2	All	80%	393	101	103	0.7955	0.7923	0.7939	2.24
nerve	Virchow2	All	85%	369	61	141	0.8581	0.7235	0.7851	1.36
nerve	Virchow2	All	90%	322	33	224	0.907	0.5897	0.7148	0.73
nerve	UNI	Colon	50%	138	425	4	0.2451	0.9718	0.3915	28.33
nerve	UNI	Colon	60%	138	302	4	0.3136	0.9718	0.4742	20.13
nerve	UNI	Colon	70%	135	259	8	0.3426	0.9441	0.5028	17.27
nerve	UNI	Colon	75%	135	213	10	0.3879	0.931	0.5477	14.2
nerve	UNI	Colon	80%	131	201	18	0.3946	0.8792	0.5447	13.4
nerve	UNI	Colon	85%	132	185	23	0.4164	0.8516	0.5593	12.33
nerve	UNI	Colon	90%	119	117	50	0.5042	0.7041	0.5877	7.8
nerve	UNI	Pancreas	50%	155	171	31	0.4755	0.8333	0.6055	11.4
nerve	UNI	Pancreas	60%	151	102	40	0.5968	0.7906	0.6802	6.8
nerve	UNI	Pancreas	70%	154	64	45	0.7064	0.7739	0.7386	4.27
nerve	UNI	Pancreas	75%	150	41	52	0.7853	0.7426	0.7634	2.73
nerve	UNI	Pancreas	80%	145	30	61	0.8286	0.7039	0.7612	2
nerve	UNI	Pancreas	85%	131	17	78	0.8851	0.6268	0.7339	1.13
nerve	UNI	Pancreas	90%	102	9	106	0.9189	0.4904	0.6395	0.6
nerve	UNI	Prostate	50%	137	155	5	0.4692	0.9648	0.6313	10.33
nerve	UNI	Prostate	60%	135	104	6	0.5649	0.9574	0.7105	6.93
nerve	UNI	Prostate	70%	137	73	6	0.6524	0.958	0.7762	4.87
nerve	UNI	Prostate	75%	136	52	6	0.7234	0.9577	0.8242	3.47
nerve	UNI	Prostate	80%	134	39	8	0.7746	0.9437	0.8508	2.6
nerve	UNI	Prostate	85%	140	19	13	0.8805	0.915	0.8974	1.27
nerve	UNI	Prostate	90%	142	2	36	0.9861	0.7978	0.882	0.13
nerve	UNI	All	50%	430	751	40	0.3641	0.9149	0.5209	16.69
nerve	UNI	All	60%	424	508	50	0.4549	0.8945	0.6031	11.29
nerve	UNI	All	70%	426	396	59	0.5182	0.8784	0.6519	8.8
nerve	UNI	All	75%	421	306	68	0.5791	0.8609	0.6924	6.8
nerve	UNI	All	80%	410	270	87	0.6029	0.8249	0.6967	6
nerve	UNI	All	85%	403	221	114	0.6458	0.7795	0.7064	4.91
nerve	UNI	All	90%	363	128	192	0.7393	0.6541	0.6941	2.84

Table S7. Ablation of SSL components for Virchow-2 and UNI on PNI detection (holdout test, n=45).

Backbone - Condition	Precision	F1	FP/Slide
Virchow-2 - Baseline	0.678	0.740	5.64
Virchow-2 - Ablation	0.766	0.804	3.80
Virchow-2 - SSL	**0.828**	**0.832**	**2.51**
UNI - Baseline	0.843	0.826	2.22
UNI - Ablation	**0.853**	0.816	**1.98**
UNI - SSL	0.788	**0.829**	3.42

Fig S1. FROC curves of Baseline vs SSL condition for UNI showing PNI (left), Nerve (centre), and Tumour (right) detection across organ types: colon (orange), pancreas (purple), prostate (green).

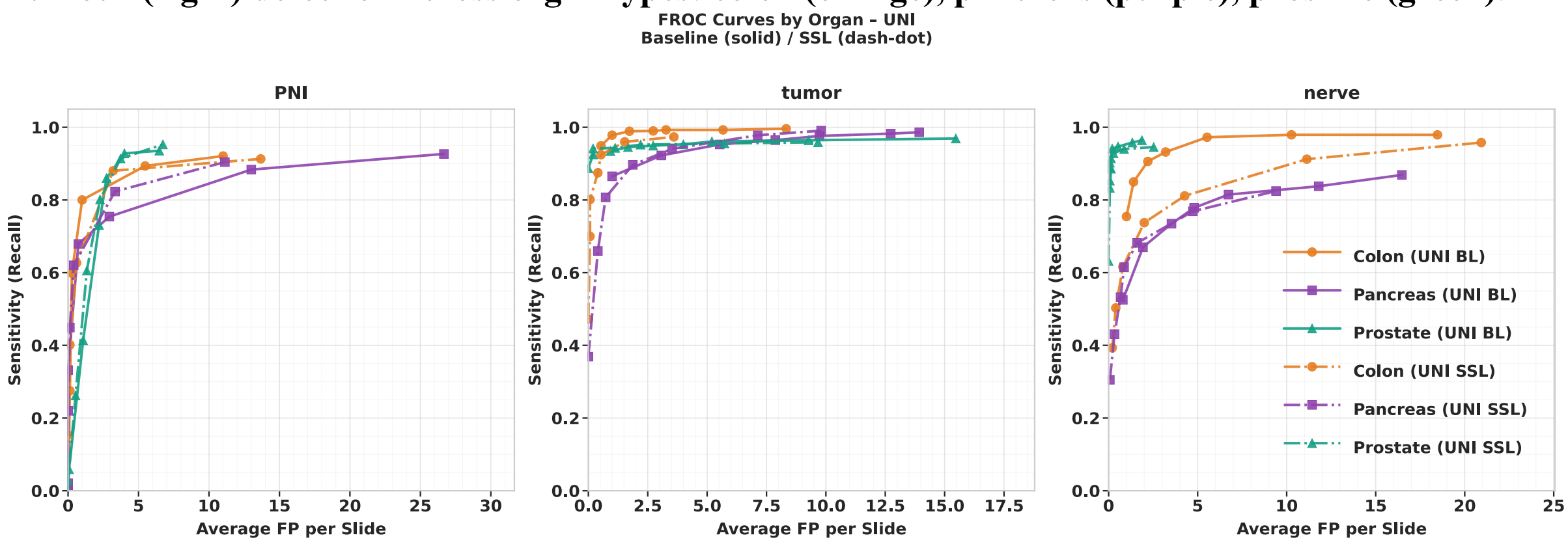

Supplement: Supplementary file 1 [file btag461_supplementary_data.pdf]
